# Polyphenols from Acorn Leaves (*Quercus liaotungensis*) Protect Pancreatic Beta Cells and Their Inhibitory Activity against α-Glucosidase and Protein Tyrosine Phosphatase 1B

**DOI:** 10.3390/molecules23092167

**Published:** 2018-08-28

**Authors:** Jing Xu, Xude Wang, Jiayin Yue, Yuanyuan Sun, Xiaoshu Zhang, Yuqing Zhao

**Affiliations:** 1 School of Functional Food and Wine, Shenyang Pharmaceutical University, Shenyang 110016, China; xj19873251@163.com (J.X.); xudewanglnu@gmail.com (X.W.); yuejiayin1990@163.com (J.Y.); sunyuanyuan921104@163.com (Y.S.); 2 Key Laboratory of Structure-Based Drug Design and Discovery of Ministry of Education, Shenyang Pharmaceutical University, Shenyang 110016, China

**Keywords:** acorn leaves, polyphenol, MIN6, pancreatic protection, α-glucosidase and PTP1B inhibitory activities

## Abstract

Acorn leaves, which possess potential pharmacologic effects, are traditionally consumed as food in China. Phytochemical investigations of acorn leaves yielded one new and 25 known polyphenols, and their structures were identified by extensive spectroscopic analysis. Three antidiabetes assays were conducted. Compound **2** considerably increased the survival of pancreatic beta cells by reducing the production of reactive oxygen species and enhancing the activities of superoxide dismutase, catalase, and glutathione in MIN6 cells damaged by H_2_O_2_. The preliminary mechanism by which compound **2** protects pancreatic beta cells was through the nuclear factor erythroid-2-related factor 2 (Nrf2)/heme oxygenase-1 HO-1 pathway. Most of the tested isolates showed strong inhibitory activity against α-glucosidase and protein tyrosine phosphatase 1B. The IC_50_ values of most compounds were much lower than those of the positive control. The results suggest that polyphenols from acorn leaves are potential functional food ingredients that can be used as antidiabetic agents.

## 1. Introduction

Diabetes mellitus is a worldwide health problem with enormous economic and social costs. The World Health Organization (WHO) predicts that approximately 300 million people (5% of the global population) will be afflicted with diabetes by 2025. Oxidative stress is closely associated with types 1 and 2 diabetes mellitus, and biomarkers of oxidative stress consistently increase in the pancreas and other tissues of patients with diabetes. Oxidative stress response could be an important mediator of damage to cell structures [1]. Therefore, prevention of oxidative stress can be used as an effective target in diabetes treatment.

Nuclear factor erythroid-2-related factor 2 (Nrf2) is a transcriptional activator that binds to the antioxidant response element of the target gene promoter [2]. Under stress conditions, Nrf2 can be transformed into the nucleus, which activates the expression of antioxidant response genes and induces detoxification in phase II. Nrf2 is considered a major regulatory factor for antioxidant resistance [3]. Heme oxygenase-1 (HO-1) is a scavenging enzyme in heme catabolism. The Nrf2/HO-1 pathway is believed to be an important scavenger in heme catabolism and plays a crucial role in protecting cells from oxidative stress [4].

α-Glucosidase, which usually exists on the brush border surface of intestinal cells, promotes the absorption of hydrolyzed polysaccharides in the small intestine and catalyzes carbohydrate dissolution [5]. Protein tyrosine phosphatase 1B (PTP1B), a member of the PTP family, functions as a negative regulator of insulin expressed ubiquitously in insulin-targeted cells. PTP1B catalyzes the dephosphorylation of activated insulin receptors and their corresponding substrate proteins [6]. Consequently, inhibitors designed to reduce the activities of α-glucosidase and PTP1B are effective in reducing blood glucose and are widely used in the treatment of type 2 diabetes [7,8].

Acorn is the fruit of the oak (*Quercus*) tree, which belongs to the Fagaceae family. Many countries produce flour, bread, and jelly made from acorn because of its rich nutrition [9]. Many researchers have reported that acorn exhibits antioxidant, antibacterial, antitumor, and antidiabetic activities [10,11]. In addition, morphological parts other than the seeds of oak tree have medical, cosmetic, or food purposes. For example, people in China use acorn leaves to wrap food for steaming. In recent years, interest in and consumption of infusions from acorn leaves have considerably increased in Mexico [12], and methods for the nanoencapsulation of tea from acorn leaves have been developed [13]. Many studies have reported about the total polyphenol, flavonoid, and proanthocyanidin content of acorn leaves [14,15], and chromatographic methods have been used to determine specific groups [16,17]. To the best of our knowledge, the active compounds and potential pharmacologic properties of acorn leaves remain unclear to date. Moreover, their nutraceutical value and functional properties have not yet been adequately studied.

Through our phytochemical investigations of acorn leaves, we isolated and determined one new flavonoid glycoside and 25 known polyphenolics by intensive nuclear magnetic resonance (NMR). Compounds **1**–**26** and the extracts were evaluated for α-glucosidase and PTP1B inhibitory activities, and their structure-activity relationships were studied. Their protective effects on pancreatic beta cells were measured in MIN6 cells damaged by H_2_O_2_. The activities of enzymes in antioxidant defense systems were further investigated. The expression levels of Nrf2 and HO-1 were detected by Western blot analysis to explore their antioxidant pathway. This study increases our knowledge about acorn leaves and promotes their potential application in functional foods.

## 2. Results and Discussion

### 2.1. Phytochemical Studies of Acorn Leaves

**Figure 1:**
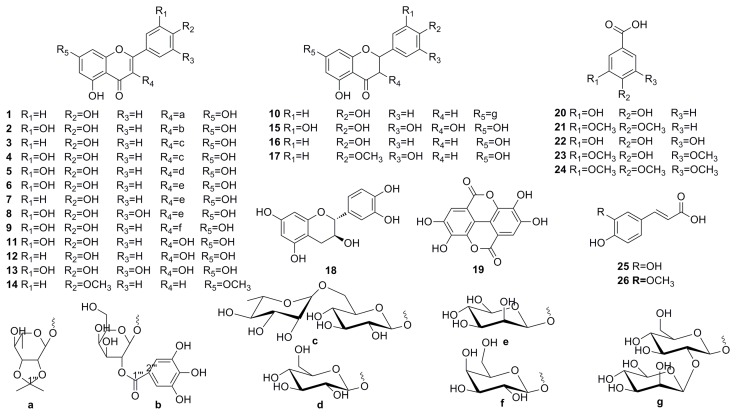
Chemical structures of isolated compounds **1**–**26**; (**a**–**g**) are the sugar groups of compounds.

Acorn leaves were extracted with 75% EtOH. Then, the extract was successively suspended in H_2_O and partitioned with petroleum ether (PE), ethyl acetate (EtOAc), and *n*-butyl alcohol (*n*-BuOH). The EtOAc soluble fractions were subjected to silica gel column chromatography (SGCC), Sephadex LH-20, MCI CC, and semipreparative HPLC to yield one new flavonoid glycoside and 25 known polyphenols (Figure 1).

**Figure 2:**
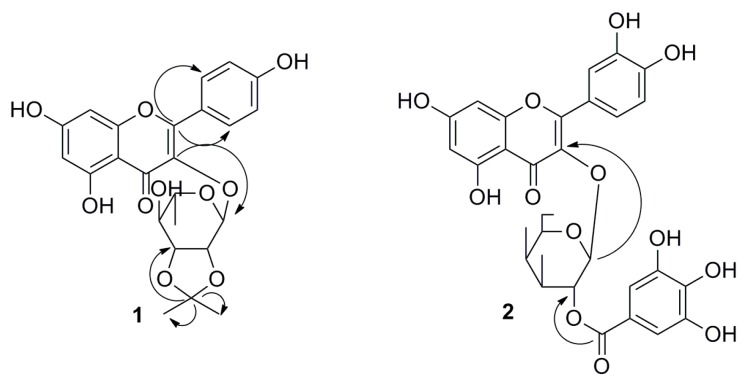
The key HMBC correlations of compounds **1**, **2**.

Compound **1** was obtained as a yellowish amorphous powder. Its molecular formula was determined to be C_24_H_24_O_10_ by HR-ESI-MS at *m*/*z* 495.1254 [M + Na]^+^ (calcd for C_24_H_24_O_10_Na, 495.1262). The ^1^H-NMR spectrum showed six signals at *δ*_H_ 6.19 (1H, d, *J* = 2.0 Hz), 6.40 (1H, d, *J* = 2.0 Hz), 6.75 (2H, d, *J* = 8.8 Hz), and 7.78 (2H, d, *J* = 8.8 Hz). This finding suggests that it is a typical trisubstituted flavonol compound. The ^13^C-NMR showed six sugar signals at *δ*c 16.5, 68.1, 72.3, 75.2, 77.8, and 97.7, as well as five sugar proton signals at *δ*_H_ 0.66 (3H, d, *J* = 6.2 Hz), 2.92–3.02 (2H, m, H-4″, 5″), 3.83 (1H, dd, *J* = 7.5, 5.8 Hz), 4.41 (1H, d, *J* = 5.8 Hz), and 5.63 (1H, br.s). Thus, the sugar was identified as rhamnose, which was linked to C-3 due to the HMBC correlation between H-1′′ (*δ*_H_, 5.63) and C-3 (*δ*c, 133.4), as shown in Figure 2. Spectral data were compared with those from kaempferol-3-*O*-α-l-rhamnoside [18], and a strong agreement was observed, with the exception of a chemical shift corresponding to C-2″, C-3″, and the presence of isopropyl group. The ^1^H-NMR spectrum of compound **1** showed signals corresponding to two tertiary methyl groups at *δ*_H_ 1.28 (3H, s) and 1.35 (3H, s), and the ^13^C-NMR showed one signal at *δ*c 108.2, which was typical of isopropyl group. The isopropyl group was further confirmed by long-range correlation observed in the HMBC spectrum among proton H-2′′′ (1.28, 3H, s), H-3′′′ (1.35, 3H, s), and carbon C-1′′′ (108.2). The linkage position of the isopropyl unit was determined through examination of HMBC cross-peak correlations between H-3″ (*δ*_H_ 3.83) and C-1′′′ (*δ*c 108.2). Therefore, compound **1** was determined to be 2″,3″-diol acetonide-3-*O*-α-l-rhamnopyranoside-kaempferol. Total assignments of protons and carbons of compound **1** were performed based on HSQC and HMBC, as shown in Table 1.

Compound **2** was obtained as a yellowish amorphous powder. Assignments of all NMR peaks based on HSQC and HMBC are presented in Table 1. The downfield part of ^1^H-NMR spectrum, namely, 6.16 (1H, d, *J* = 1.4 Hz), 6.33 (1H, d, *J* = 1.4 Hz), 6.78 (1H, d, *J* = 8.5 Hz), 7.49 (1H, d, *J* = 8.5, 1.6 Hz), and 7.64 (1H, d, *J* = 1.6 Hz), well corresponded to the structure of quercetin. A singlet at 7.13 ppm, which was assigned to the gallic acid moiety, was also observed in the downfield area of the proton spectrum. Signals in the area from 3.68 to 5.68 were assigned to sugar moiety. Additionally, five carbons at *δ*c 62.1, 70.5, 73.5, 74.6, 77.5, and 101.2 were observed; thus, the sugar was identified as galactose. The anomeric proton of sugar was observed at 5.68 (1H, d, *J* = 7.9 Hz). The sugar was assumed to be in β configuration because the doublet had a *J* value of 7.9 Hz. A long-range correlation in the HMBC spectrum between the H-1″ of galactose (5.76) and the C-3 of aglycone (135.1) confirmed that the galactose was linked at C-3 of the flavonol skeleton. Furthermore, a long-range correlation between H-2″ (5.44) of sugar and C-1′′′ (168.2) confirmed that galloyl moiety was attached to C-2″ of sugar (Figure 2). Consequently, compound **2** was identified as quercetin-3-*O*-(2″-*O*-galloyl)-β-galactopyranoside. ^1^H-NMR and ^13^C-NMR data well corresponded to that previously reported for the compound (Pakulski & Budzianowski, 1996) [19].

Other known compounds (see Figure 1) were characterized by their corresponding NMR spectra (Supplementary Figures S10–S56). These compounds were identified as follows: kaempferol-3-*O*-rutinoside (**3**), quercetin-3-*O*-rutinoside (**4**), isoquercitrin (**5**), quercetin-3-*O*-α-l-rhamnopyranoside (**6**), kaempferol-3-*O*-α-l-rhamnoside (**7**), myricetin-3-*O*-α-l-rhamnopyranoside (**8**), quercetin-3-*O*-galactoside (**9**), 7-rhamnosidoglucoside-2,3-dihydro-4′,5,7-trihydroxyflavone (**10**), quercetin (**11**), kaempferol (**12**), myricetin (**13**), 5-hydroxyl-7,4′-dimethoxy flavone (**14**), dihydromyricetin (**15**), 4′,5,7-trihydroxyflavanone (**16**), 4′-methoxy-5′,5,7-trihydroxyflavanone (**17**), catechin (**18**), ellagic acid (**19**), protocatechuic acid (**20**), 3,4-dimethoxybenzoic acid (**21**), gallic acid (**22**), syringic acid (**23**), 3,4,5-trimethoxybenzoic acid (**24**), caffeic acid (**25**), and ferulic acid (**26**).

### 2.2. Protective Effects on Pancreatic Beta Cells (MIN6) Damaged by H_2_O_2_

**Figure 3:**
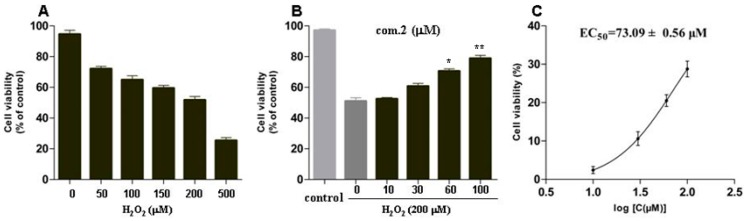
Protective effects of compound **2** against H_2_O_2_-induced cell oxidative stress damage in MIN6 cells. (**A**) Cells were treated with increasing concentrations of H_2_O_2_ for 12 h. (**B**) Cells were treated with various concentrations of compound **2** for 4 h, and then exposed to 200 μM H_2_O_2_ for 12 h. (**C**) EC_50_ of compound **2** on H_2_O_2_-induced MIN6 cells. Data are presented as means ± standard deviations (SDs) of three independent experiments. * *p* < 0.05, ** *p* < 0.01, compared with com.2 (0 μM).

MIN6 cells were treated with H_2_O_2_ at different concentrations (0, 50, 100, 150, 200, and 500 μM) for 12 h. Cell viability was measured by the MTT method. As shown in Figure 3, the viability of MIN6 cells treated with 200 μM H_2_O_2_ was considerably lower than that of the untreated group, and the cell viability was 51.73 ± 3.99%. Therefore, the cell model of oxidative damage was established by treating with 200 μM H_2_O_2_ for 12 h.

Then, the protective effects of compounds **1**–**26** on oxidative stress–induced injury in MIN6 cells were also tested (Table 2). Cells were treated with various concentrations of compounds **1**–**26** for 4 h and then exposed to 200 μM H_2_O_2_ for 12 h. Among them, compound **2** showed a substantial protective effect on the damaged MIN6 cells, with an EC_50_ value of 73.09 ± 0.56 μM (Figure 3B,C). The preliminary structure–activity relationship of all compounds indicated that most of the flavonoid glycosides and phenolic acids did not express considerable biological activity. The results showed that the flavonoids exhibited stronger protection than their flavonoid glycosides. However, flavonoids with galloyl showed stronger protective effects than those with hydroxyl. Notably, the substituted galloyl (such as compound **2**) is necessary for the bioactivity on the basis of preliminary screening results.

### 2.3. Compound ***2*** Enhanced the Activities of SOD, CAT, and GSH in Pancreatic Beta Cells (MIN6) Damaged by H_2_O_2_

**Figure 4:**
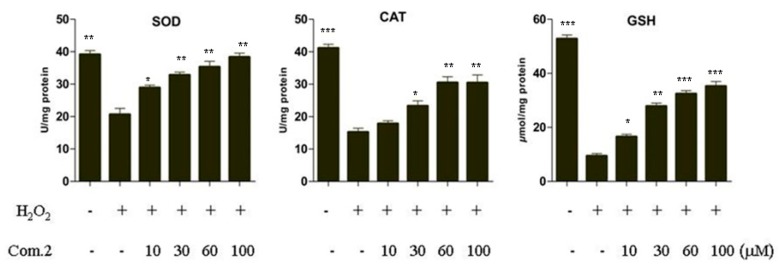
Compound **2** restored superoxide dismutase (SOD) and catalase (CAT) activities and glutathione (GSH) level in MIN6 cells damaged by H_2_O_2_. Cells were treated with various concentrations of compound **2** for 4 h, then exposed to 200 μM H_2_O_2_ for 12 h. SOD, CAT, and GSH were measured with microplate. Data are presented as means ± SDs of three independent experiments. * *p* < 0.05, ** *p* < 0.01, *** *p* < 0.001 compared with com.2 (0 μM) in 200 μM H_2_O_2_.

Superoxide dismutase (SOD) and catalase (CAT) are antioxidant enzymes that play important roles in preventing oxidative injury. Glutathione (GSH) is a major tissue antioxidant maintained in reduced form by glutathione reductase, and it provides reducing equivalents for glutathione peroxidase catalyzing reduction [20]. The activities of SOD, CAT, and GSH in MIN6 cells damaged by H_2_O_2_ were measured to evaluate the effects of compound **2** on antioxidant defense systems. The results showed that their activities in MIN6 cells damaged by H_2_O_2_ were considerably lower than those in the control group. However, treatment with compound **2** markedly increased the activities of these enzymes compared with those of the H_2_O_2_-damaged group (Figure 4). These data suggest that compound **2** demonstrates strong antioxidative effects on MIN6 cells damaged by H_2_O_2_.

### 2.4. Compound ***2*** Reduced the Amount of Intracellular ROS

**Figure 5:**
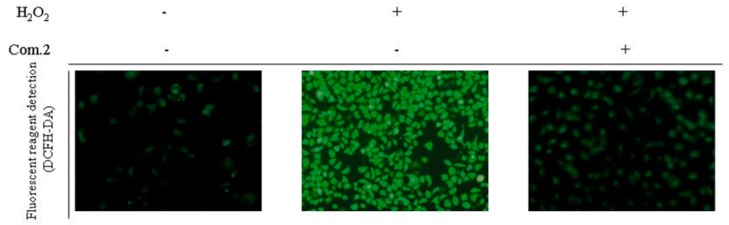
Compound **2** reduced the amount of intracellular reactive oxygen species (ROS). Cells were treated with 100 μM of compound **2** for 4 h, then exposed to 200 μM H_2_O_2_ for 12 h. Cells were stained with dichloro-fluorescein-diacetate (DCFH-DA). The DCF fluorescence intensity was measured by fluorescence microscope.

Oxidative stress response is often accompanied by the production of a large number of reactive oxygen species (ROS). H_2_O_2_ induces ROS production imbalance and destroys MIN6 cells, resulting in oxidative stress damage. Dichloro-fluorescein-diacetate (DCFH-DA) fluorescence probe was used to detect the level of intracellular ROS in MIN6 cells damaged by H_2_O_2_ and investigate whether compound **2** plays a protective role by inhibiting ROS production. Nonfluorescent DCFH-DA dye can freely enter the cell membrane and hydrolyze to form DCFHs by intracellular esterase. In the presence of ROS, DCFHs were rapidly oxidized to form highly fluorescent DCFs. The fluorescence intensity of DCFs is assumed to be parallel to the amount of ROS formed in cells. The fluorescence intensity of DCFs in the H_2_O_2_-damaged group was considerably higher than in the control group. Meanwhile, treatment with 100 μM of compound **2** eliminated intracellular ROS in the oxidative stress injury of H_2_O_2_-damaged group, suggesting that compound **2** can protect MIN6 cells from excessive ROS damage (Figure 5).

### 2.5. Compound ***2*** Activated Nrf2/HO-1 Pathway

**Figure 6:**
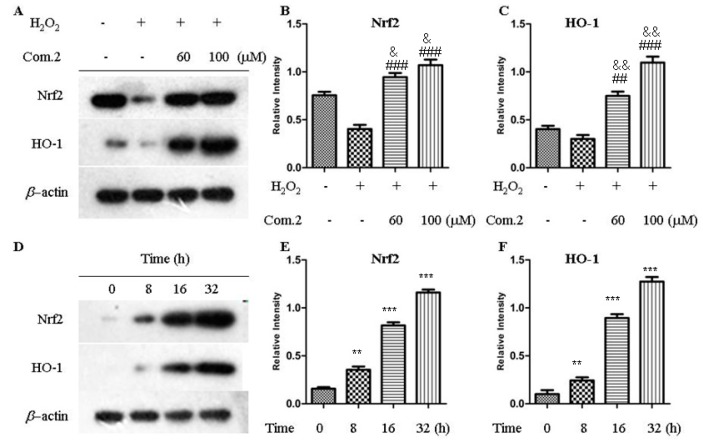
Effects of compound **2** on the expression of Nrf2 and HO-1 in MIN6 cells. (**A**) Expression levels of Nrf2-mediated antioxidant proteins were detected by Western blot analysis in 16 h. (**B**,**C**) Quantification of Nrf2 and HO-1 protein expression. Data are presented as means ± SDs of three independent experiments. ^&^ *p* < 0.05, ^&&^ *p* < 0.01 compared with control group (untreated with H_2_O_2_ and compound **2**). ^##^ *p* < 0.01, ^###^ *p* < 0.001 compared with model group (only treated with H_2_O_2_). (**D**) Expression levels of Nrf2-mediated antioxidant proteins after the cells were incubated with 100 μM of compound **2** for the indicated time period. (**E**,**F**) Quantification of Nrf2 and HO-1 protein expression. Data are presented as means ± SDs of three independent experiments. ** *p* < 0.01, *** *p* < 0.001 compared with control group.

Nrf2 is a stress-response transcriptional activator that protects cells from oxidative damage. Under oxidative stress, Nrf2 was transferred into the nucleus to induce the expression of HO-1 [21]. Thus, the effects of compound **2** on the expression levels of Nrf2 and HO-1 were tested. The result showed that treatment of compound **2** with 60 and 100 μM can substantially increase the expression of Nrf2 and HO-1. Furthermore, Western blot analysis revealed that the expression of Nrf2 and HO-1 increased in a time-dependent manner (Figure 6).

### 2.6. PTP1B and α-Glucosidase Inhibition Assay

Natural inhibition of PTP1B and α-glucosidase may be the main way to develop antidiabetic drugs. Phenolic and flavone compounds have been recognized as PTP1B and α-glucosidase inhibitors, respectively. A total of 26 compounds from the extract, including 10 flavonoid glycosides, 8 flavonoids, and 8 phenolic acids, were tested for their PTP1B and α-glucosidase inhibition activities. The IC_50_ values of the two inhibitions are shown in Table 2.

The extract of acorn leaves demonstrated strong inhibitory activity against PTP1B, with an IC_50_ value of 40.16 μg/mL. Compounds **11**, **12**, **13**, **15**, **16**, **17**, and **19** showed considerable PTP1B inhibitory activity at IC_50_ values of 4.16, 3.92, 3.53, 9.58, 15.38, 20.16, and 1.03 µM, respectively, compared with the IC_50_ of the positive control Na_3_VO_4_ at 28.91 µM. Meanwhile, the results showed that the flavonoids exhibited stronger activity than the flavonoid glycosides. Among the flavonoid glycosides, only compound **2** substituted with galloyl showed much stronger activity than the other flavonoid glycosides, which do not have a galloyl group. On the basis of these experimental data, the relationship between structure and activity may be related to the hydroxyl group. The activities of the compounds with hydroxyl were higher than those with methoxyl, and the inhibitory activity weakened when the hydroxyl was substituted for sugar.

As shown in Table 2, the extract of acorn leaves exhibited potent inhibition of α-glucosidase, with an IC_50_ value of 15.63 μg/mL. Compounds **11**, **12**, and **13** considerably inhibited α-glucosidase enzyme at IC_50_ values of 1.69, 2.73, and 0.52 µM, respectively, compared with the IC_50_ of the positive control acarbose at 5.90 µM. The different inhibitions against α-glucosidase were possibly associated with their different structure types. Of all the tested compounds, the flavonoids demonstrated higher inhibitory activity than the flavonoid glycosides. Consistent with the results obtained for PTP1B inhibition determined in this study, the same trend of α-glucosidase inhibition was observed in compound **2**, which showed much stronger activity than the other flavonoid glycosides that do not possess a galloyl group. The IC_50_ value of compound **13** with three hydroxyl groups in ring B was lower than those of **11** and **12**. Therefore, the relationship between structure and activity may be associated with the hydroxyl, and the hydroxyl on ring B may strengthen the inhibitory activity.

Diabetes mellitus, as a metabolic disorder, is a complex chronic disease related to a variety of targets. Different targets were combined with various compounds, such as PTP1B and α-glucosidase, which provide an opportunity for the treatment of diabetes.

## 3. Materials and Methods

### 3.1. Reagents and Chemicals

Silica gel (mesh: 300–400) from Qingdao Marine Chemistry Co. (Qingdao, China), RP C18 silica gel (mesh: 300–400) from Agela Technology (Tianjin, China), Sephadex LH-20 from Amersham (Uppsala, Sweden), and MCI Gel CHP20P (70–150 μM) from Mitsubishi (Tokyo, Japan) were procured. A semipreparative HPLC system (CXTH LC3000, Beijing, China) and an octadecyl silica chromatographic column (YMC-Pack ODS A; 5 mm, 250 mm × 10 mm) were used. The flow rate of the semipreparative HPLC was 3.0 mL/min, and the HPLC column temperature was 25 °C. The ultraviolet spectrophotometric detection of semipreparative HPLC was 203 nm. Purified water was purchased from Wahaha (Hangzhou, China). Methanol, formic acid (HPLC grade), and other solvents (analytical grade) were purchased from Kangkede (Tianjin, China). PTP1B (human, recombinant), yeast α-glucosidase, dithiothreitol (DTT), *p*-nitrophenyl phosphate (pNPP), *p*-nitrophenyl-α-d-glucopyranoside (pNPG), and β-mercaptoethanol were purchased from Sigma-Aldrich (St. Louis, MO, USA). Other chemicals (analytical grade) were purchased from Sinopharm. Superoxide dismutase (SOD), catalase (CAT), glutathione (GSH), and protein assay kits were obtained from Nanjing Jiancheng Bioengineering Institute (Nanjing, China). Primary antibodies against rabbit Nrf2, HO-1, and secondary horseradish peroxidase (HRP)-labeled goat anti-rabbit antibodies were purchased from Abcam (Cambridge, UK). Antibody against rabbit β-actin was purchased from Bioworld Technology (Nanjing, China). NMR spectra were recorded with a Bruker Avance spectrometer (Bruker Co., Karlsruhe, Germany) with tetramethylsilane as the internal standard.

### 3.2. Plant Material

Leaves identified as those from *Quercus liaotungensis* by Prof. Jincai Lu (Shenyang Pharmaceutical University, Shenyang, China) were collected from Liaoning, China, in June 2015. A voucher specimen (XY. 201506) was deposited in the Department of Natural Products Chemistry, Shenyang Pharmaceutical University.

### 3.3. Extraction and Isolation

Dried acorn leaves (3.0 kg) were extracted with 75% EtOH (20.0 L × 3, 2 h each) under reflux, filtered, and then concentrated to yield 5.0 L of aqueous residue to obtain the active constituents. The aqueous residue (5.0 L) was partitioned with petroleum ether (PE), ethyl acetate (EtOAc), and *n*-butyl alcohol (*n*-BuOH) (5.0 L × 3 in each case), and the yields of their extracts were 40.0, 90.0, and 160.0 g, respectively. The EtOAc extract (90.0 g) was chromatographed over a silica gel column (10 × 80 cm) eluted with CH_2_Cl_2_/CH_3_OH (1:0 to 0:1) to obtain 10 fractions (A1–A10). Fraction A8 (8.0 g) was subjected to an MCI column eluted with CH_3_OH/H_2_O (30:70 to 100:0) to yield A81–A89. Subfraction A82 (2.0 g) was chromatographed over silica gel column chromatography (SGCC) eluted with CH_2_Cl_2_/CH_3_OH (1:0 to 0:1) to yield compounds **3** (33.2 mg) and **4** (37.3 mg). Subfraction A83 was separated by HPLC eluted with 45% CH_3_OH/H_2_O to obtain compounds **10** (5.4 mg, t_R_ = 21 min) and **2** (6.3 mg, t_R_ = 29 min). Subfraction A84 was subjected to a Sephadex LH-20 column and then purified by HPLC (55% CH_3_OH/H_2_O) to yield compounds **9** (7.1 mg, t_R_ = 31 min) and **5** (8.2 mg, t_R_ = 37 min). Subfraction A85 was separated by HPLC eluted with 60% CH_3_OH/H_2_O to acquire compound **8** (5.9 mg, t_R_ = 26 min). Subfraction A86 was subjected to a Sephadex LH-20 column and then purified by HPLC (65% CH_3_OH/H_2_O) to obtain compounds **6** (6.1 mg, t_R_ = 27 min) and **7** (6.8 mg, t_R_ = 38 min). Fraction A7 was subjected to an MCI column eluted with CH_3_OH/H_2_O (30:70 to 100:0) to yield A71–A75. Subfraction A73 was chromatographed over a Sephadex LH-20 column and then purified by HPLC (65% CH_3_OH/H_2_O) to acquire compound **1** (5.9 mg, t_R_ = 45 min). A74 was chromatographed over a Sephadex LH-20 column, and fraction A746 was purified by HPLC (70% CH_3_OH/H_2_O) to yield compounds **15** (8.7 mg, t_R_ = 31 min) and **13** (7.7 mg, t_R_ = 39 min). A748 was purified by HPLC (73% CH_3_OH/H_2_O) to produce compounds **18** (4.7 mg, t_R_ = 29 min) and **19** (7.1 mg, t_R_ = 41 min). A6 (2.0 g) was chromatographed over SGCC eluted with CH_2_Cl_2_/CH_3_OH (1:0 to 0:1) to generate compounds **11** (27.3 mg) and **12** (33.6 mg). A5 (6.0 g) was subjected to an MCI column eluted with CH_3_OH/H_2_O (30:70 to 100:0) to produce 6 fractions (A51–A56). Subfraction A53 was chromatographed over a Sephadex LH-20 column (CH_2_Cl_2_:CH_3_OH = 1:1) and then separated by HPLC (73% CH_3_OH/H_2_O) to yield compounds **16** (6.0 mg, t_R_ = 36 min) and **17** (6.6 mg, t_R_ = 50 min). Subfraction A54 was chromatographed over a Sephadex LH-20 column (CH_2_Cl_2_:CH_3_OH = 1:1) and then separated by HPLC (75% CH_3_OH/H_2_O) to yield compound **14** (6.2 mg, t_R_ = 33 min). Fraction A3 (7.0 g) was chromatographed over SGCC eluted with PE/EtOAc (1:0 to 0:1) to obtain 6 fractions (A31–A36). Fraction A35 was chromatographed over a Sephadex LH-20 column (CH_2_Cl_2_:CH_3_OH = 1:1) and then separated by HPLC (76% CH_3_OH/H_2_O) to yield compounds **22** (12.6 mg, t_R_ = 36 min) and **20** (6.2 mg, t_R_ = 44 min). Fraction A34 was chromatographed over a Sephadex LH-20 column (CH_2_Cl_2_:CH_3_OH = 1:1) and then separated by HPLC (78% CH_3_OH/H_2_O) to yield compounds **25** (5.6 mg, t_R_ = 31 min) and **26** (5.9 mg, t_R_ = 40 min). Fraction A33 was chromatographed over a Sephadex LH-20 column (CH_2_Cl_2_:CH_3_OH = 1:1) and then separated by HPLC (85% CH_3_OH/H_2_O) to obtain compounds **23** (5.7 mg, t_R_ = 33 min) and **21** (5.5 mg, t_R_ = 42 min). Finally, compound **24** (33.2 mg) was purified by an MCI column eluted with CH_3_OH/H_2_O (30:70 to 100:0) from subfraction A32.

### 3.4. Cell Culture and Cell Viability Assay

Mouse normal pancreatic beta cell line MIN6 was obtained from Beijing Dingguo Biotechnology Co., Ltd. (Beijing, China). The cells were cultured in RAPI-1640 medium (HyClone, Thermo Scientific, Waltham, MA, USA) supplemented with 10% heat-inactivated fetal bovine serum and then maintained at 37 °C with 5% CO_2_ in a humidified atmosphere. Cell viability was measured using the MTT assay. Cells (6 × 10^4^ per mL) were cultured in a 96-well (100 μL per well) microplate at 37 °C in humidified 5% CO_2_ for 24 h. Cells were also treated with various concentrations of compounds for 4 h and then exposed to 200 μM H_2_O_2_ for 12 h. The absorptions were measured at 490 nm with a microplate reader (iMark, Bio-Rad, Hercules, CA, USA).

### 3.5. Determining CAT, SOD, and GSH

CAT and SOD as antioxidant enzymes and nonenzymatic antioxidant (GSH) in pancreatic cells were determined using commercial kits in accordance with their guidelines. The absorbance of each well was measured by a microplate reader (iMark, Bio-Rad, Hercules, CA, USA).

### 3.6. Determining the Amount of Intracellular ROS

The amount of intracellular ROS was measured by the fluorescence probe-2′,7′-dichloro-fluorescein-diacetate (DCFH-DA) purchased from Sigma. MIN6 cells were cultured in 6-well plates for 24 h and then incubated with H_2_O_2_ or a combination of H_2_O_2_ and compound **2** for 12 h. The cells were treated with 10 μM DCFH-DA and incubated in darkness for 30 min. Then, the fluorescence intensity was measured under a fluorescence microscope.

### 3.7. Western Blot Analysis

We used RIPA lysis buffer to extract total cellular proteins, and the protein concentration was measured by bicinchoninic acid. SDS-PAGE was conducted in 10% gel, and the protein load in each lane was equal. The resolved protein bands were transferred onto polyvinylidene difluoride (PVDF) membranes after electrophoresis, and the membranes were blocked with 5% bovine serum albumin (BSA) in TBST buffer for 1 h. The PVDF membranes were incubated with primary antibodies against Nrf2 and HO-1 (1:1000) in 5% BSA at 4 °C overnight, washed with TBST containing 0.1% Tween-20, and then incubated with HRP-conjugated secondary antibody (1:5000) at room temperature for 2 h. The enhanced chemiluminescence system was used to display positive bands on X-ray film.

### 3.8. Assay for PTP1B Inhibitory Activity

The PTP1B inhibition assay was performed as previously reported [22]. Exactly 83 µL of the enzyme in buffer (pH 7.5) consisting of 2 mM β-mercaptoethanol, 25 mM Tris-HCl, 1 mM DTT, 1 mM EDTA, and 10 µL of different concentrations of compounds were incubated in a 96-well plate at 37 °C for 10 min. Subsequently, the enzyme was incubated with the substrate pNPP (4 µL, 10 mM) in a buffer at 37 °C for 30 min, and NaOH (5 µL, 2 mol/L) was added to stop the reaction. In the blank control group, 1% DMSO (10 µL) was added to replace the sample solution. The dephosphorylation product pNP can be monitored at 405 nm. Therefore, the absorbance of the sample was obtained at 405 nm with a microplate reader.

### 3.9. Assay for α-Glycosidase Inhibitory Activity

A slightly modified and developed method of the α-glucosidase assay was used [23]. α-Glycosidase solution (30 µL, in 0.1 mol/L potassium phosphate buffer, 2 units/mL, pH 6.8) was mixed with different concentrations of compounds (20 µL, in 1% DMSO) and incubated at 37 °C for 5 min. Then, pNPG (150 µL, 10 mM) and potassium phosphate buffer (800 µL, 0.1 M) were added to the solution. After 30 min of incubation at 37 °C, the reaction was stopped by adding Na_2_CO_3_ (2 mL, 1 M). In the blank control group, 1% DMSO (20 μL) was added instead of the sample solution. The absorbance of the released product (*p*-nitrophenol) was obtained at 405 nm with a microplate reader.

### 3.10. Statistical Analysis

All data represent the mean ± standard deviation (SD) of three independent experiments. Two-way ANOVA was performed using Prism 5.0 (GraphPad Software Inc., San Diego, CA, USA). The intensity of Western blot bands was analyzed by ImageJ, and statistical analyses were conducted using SPSS software package (version 17.0, IBM Corp., Armonk, NY, USA).

## 4. Conclusions

One new flavonoid glycoside and 25 polyphenolics were isolated from acorn leaves. The protective effects on normal pancreatic beta cells damaged by H_2_O_2_ and the inhibitory activities of α-glucosidase and PTP1B were tested, which are directly related to the absorption or metabolism of glucose. Among them, compound **2** demonstrated the most potent protective effects on MIN6 cells damaged by H_2_O_2_ oxidative stress. This compound improved cell survival by decreasing ROS and enhancing the activities of SOD, CAT, and GSH, which can avoid the oxidative injury of MIN6 cells damaged by H_2_O_2_. In addition, compound **2** activated the Nrf2/HO-1 pathway in MIN6 cells damaged by H_2_O_2_ oxidative stress. Consequently, compound **2** exerted antioxidative effects on damaged pancreas, suggesting its potential for development into a protective or therapeutic drug for the pancreas.

Moreover, the acorn leaf extracts and most of the polyphenolics showed strong inhibitory activity against α-glucosidase and PTP1B. The influence of minor structural differences was also observed on the inhibitory activity, which may provide a basis for the development of new hypoglycemic agents. Overall, this study provides a foundation for future investigation of the potential hypoglycemic function of acorn leaves and the development of dietary supplements for prevention of diabetes mellitus.

## Figures and Tables

**Table 1:** NMR data of compounds **1**, **2** (^1^H: 600 MHz, ^13^C: 150 MHz), *δ* in ppm ^a^.

Position	1		Position	*2*	
	*δ*_c_	*δ*_H_, (*J* in Hz)		*δ*_c_	*δ*_H_, (*J* in Hz)
2	157.2		2	158.1	
3	133.4		3	135.1	
4	177.5		4	179.1	
5	161.2		5	163.1	
6	98.8	6.19, d (2.0)	6	99.7	6.16, d (1.4)
7	164.4		7	165.7	
8	93.8	6.40, d (2.0)	8	94.5	6.33, d (1.4)
9	156.5		9	158.3	
10	104.0		10	105.8	
1′	120.3		1′	123.1	
2′	130.6	7.78, d (8.8)	2′	117.2	7.64, d (1.6)
3′	115.5	6.75, d (8.8)	3′	145.9	
4′	160.2		4′	149.7	
5′	115.5	6.75, d (8.8)	5′	116.2	6.78, d (8.5)
6′	130.6	7.78, d (8.8)	6′	123.0	7.49, dd (8.5, 1.6)
1″ (Rha)	97.7	5.63, br.s	1″ (Gal)	101.2	5.68, d (7.9)
2″	75.2	4.41, d (5.8)	2″	74.6	5.44, t-like
3″	77.8	3.83, dd (5.8, 7.5)	3″	73.5	3.82, dd (3.3, 9.9)
4″	68.1	3.02 m	4″	70.5	3.92, d (3.2)
5″	72.3	2.92 m	5″	77.5	3.59, t-like
6″	16.5	0.66, d (6.2)	6″	62.1	3.68, m
1‴	108.2		1‴ (galloyl)	168.2	
2‴	26.2	1.28 s	2‴	121.6	
3‴	27.9	1.35 s	3‴, 7‴	110.6	7.13, s
–	–	–	4‴, 6‴	146.3	
–	–	–	5‴	139.8	

^a^ Compound **1** was measured in DMSO-*d*_6_, **2** was measured in CD_3_OD.

**Table 2:** Cell viability and inhibitory activities of extracts and isolated compounds. PTP1B, protein tyrosine phosphatase 1B.

Compound	Cell Viability	PTP1B	α-Glucosidase
	% of Control ^a^	IC_50_	IC_50_
**1**	50.49 ± 1.68	80.19 ± 5.28 ^b^	60.52 ± 4.19 ^b^
**2**	81.52 ± 2.66	5.56 ± 0.38 ^b^	8.59 ± 1.52 ^b^
**3**	56.22 ± 3.98	24.89 ± 2.39 ^b^	30.06 ± 3.19 ^b^
**4**	59.27 ± 4.83	20.56 ± 2.01 ^b^	25.33 ± 5.19 ^b^
**5**	55.13 ± 2.78	82.87 ± 8.61 ^b^	53.98 ± 3.09 ^b^
**6**	57.19 ± 4.68	81.59 ± 7.09 ^b^	52.09 ± 4.65 ^b^
**7**	55.21 ± 3.19	98.01 ± 8.21 ^b^	59.11 ± 5.06 ^b^
**8**	59.98 ± 4.61	70.52 ± 5.25 ^b^	49.37 ± 5.21 ^b^
**9**	58.99 ± 3.68	78.42 ± 6.15 ^b^	52.17 ± 5.91 ^b^
**10**	53.29 ± 2.76	89.56 ± 7.06 ^b^	69.38 ± 5.81 ^b^
**11**	65.35 ± 5.35	4.16 ± 0.56 ^b^	1.61 ± 0.06 ^b^
**12**	63.77 ± 4.15	3.92 ± 0.36 ^b^	2.73 ± 0.33 ^b^
**13**	67.29 ± 3.16	3.53 ± 0.92 ^b^	0.52 ± 0.09 ^b^
**14**	70.17 ± 3.26	>100 ^b^	>100 ^b^
**15**	69.71 ± 3.07	9.58 ± 1.82 ^b^	7.56 ± 1.99 ^b^
**16**	63.29 ± 3.18	15.38 ± 2.76 ^b^	10.96 ± 1.78 ^b^
**17**	72.27 ± 4.69	20.16 ± 3.02 ^b^	15.28 ± 1.77 ^b^
**18**	57.66 ± 4.56	51.11 ± 5.92 ^b^	63.93 ± 5.12 ^b^
**19**	68.51 ± 3.78	1.03 ± 0.12 ^b^	9.45 ± 1.62 ^b^
**20**	53.26 ± 4.13	81.16 ± 7.39 ^b^	45.08 ± 3.52 ^b^
**21**	49.79 ± 3.98	>100 ^b^	>100 ^b^
**22**	55.49 ± 4.49	79.07 ± 7.99 ^b^	43.58 ± 5.09 ^b^
**23**	51.22 ± 3.10	>100 ^b^	>100 ^b^
**24**	50.62 ± 2.91	>100 ^b^	>100 ^b^
**25**	52.56 ± 3.58	50.19 ± 6.01 ^b^	60.09 ± 5.29 ^b^
**26**	52.96 ± 2.86	70.99 ± 7.19 ^b^	90.89 ± 9.69 ^b^
75% EtOH extract	64.16 ± 5.06	40.16 ± 4.53 ^c^	15.63 ± 2.11 ^c^
Na_3_VO_4_	–	28.91 ± 2.78 ^b^ 5.32 ± 0.51 ^c^	
Acarbose	–		5.90 ± 0.98 ^b^ 3.81 ± 0.63 ^c^

^a^ Cell protection rate of the compounds (100 μM) and the extract (100 μg/mL) on H_2_O_2_-treated MIN6 cells. ^b^ μM. ^c^ μg/mL.

## Supplementary Materials

Spectroscopic data of compounds **1**–**26**.
