# Supplementary material for: Polyphenols from Acorn Leaves (Quercus liaotungensis) Protect Pancreatic Beta Cells and Their Inhibitory Activity against α-Glucosidase and Protein Tyrosine Phosphatase 1B
Source: Molecules. 2018 Aug 28;23(9):2167. doi: 10.3390/molecules23092167 (PMC6225166; doi:10.3390/molecules23092167)
Supplement: Supplementary file 1 [file molecules-23-02167-s001.pdf]

Supplementary Materials

Polyphenols from acorn leaves (Quercus liaotungensis) protect pancreatic beta cells and their inhibitory activity against *α*-glucosidase and protein tyrosine phosphatase 1B

Jing Xu[a,b], Xude Wang[a,b], Jiayin Yue[a,b], Yuanyuan Sun[a,b], Xiaoshu Zhang[a,b*], Yuqing Zhao[a, b*]

[a] *School of Functional Food and Wine, Shenyang Pharmaceutical University, Shenyang 110016, People's Republic of China.*

[b] *Key Laboratory of Structure-based Drug Design and Discovery of Ministry of Education, Shenyang Pharmaceutical University, Shenyang 110016, China.*

* Corresponding author.

Tel: +86-24-23986521, Fax: +86-24-23986521, email: zyq4885@126.com *(Y. Zhao) or Tel: +86-24-23986522, Fax: +86-24-23986521, email: xiaoshu2397@163.com *(X. Zhang)

CONTENT

Figure S1. HR-ESI-MS of compound 1

Figure S2. ^{1}H-NMR of Compound 1

Figure S3. ^{13}C-NMR of Compound 1

Figure S4. HSQC of Compound 1

Figure S5. HMBC of Compound 1

Figure S6. ^{1}H-NMR of of compound 2

Figure S7. ^{13}C-NMR of Compound 2

Figure S8. HSQC of Compound 2

Figure S9. HMBC of Compound 2

Figure S10. ^{1}H-NMR of Compound 3

Figure S11. ^{13}C-NMR of Compound 3

Figure S12. ^{1}H-NMR of Compound 4

Figure S13. ^{13}C-NMR of Compound 4

Figure S14. ^{1}H-NMR of Compound 5

Figure S15. ^{13}C-NMR of Compound 5

Figure S16. ^{1}H-NMR of Compound 6

Figure S17. ^{13}C-NMR of Compound 6

Figure S18. ^{1}H-NMR of Compound 7

Figure S19. ^{13}C-NMR of Compound 7

Figure S20. ^{1}H-NMR of Compound 8

Figure S21. ^{13}C-NMR of Compound 8

Figure S22. ^{1}H-NMR of Compound 9

Figure S23. ^{13}C-NMR of Compound 9

Figure S24. ^{1}H-NMR of Compound 10

Figure S25. ^{13}C-NMR of Compound 10

Figure S26. ^{1}H-NMR of Compound 11

Figure S27. ^{13}C-NMR of Compound 11

Figure S28. ^{1}H-NMR of Compound 12

Figure S29. ^{13}C-NMR of Compound 12

Figure S30. ^{1}H-NMR of Compound 13

Figure S31. ^{13}C-NMR of Compound 13

Figure S32. ^{1}H-NMR of Compound 14

Figure S33. ^{13}C-NMR of Compound 14

Figure S34. ^{1}H-NMR of Compound 15

Figure S35. ^{13}C-NMR of Compound 15

Figure S36. ^{1}H-NMR of Compound 16

Figure S37. ^{13}C-NMR of Compound 16

Figure S38. ^{1}H-NMR of Compound 17

Figure S39. ^{13}C-NMR of Compound 17

Figure S40. ^{1}H-NMR of Compound 18

Figure S41. ^{13}C-NMR of Compound 18

Figure S42. ^{1}H-NMR of Compound 19

Figure S43. ^{13}C-NMR of Compound 19

Figure S44. ^{1}H-NMR of Compound 20

Figure S45. ^{13}C-NMR of Compound 20

Figure S46. ^{1}H-NMR of Compound 21

Figure S47. ^{13}C-NMR of Compound 21

Figure S48. ^{1}H-NMR of Compound 22

Figure S49. ^{13}C-NMR of Compound 22

Figure S50. ^{1}H-NMR of Compound 23

Figure S51. ^{13}C-NMR of Compound 23

Figure S52. ^{13}C-NMR of Compound 24

Figure S53. ^{1}H-NMR of Compound 25

Figure S54. ^{13}C-NMR of Compound 25

Figure S55. ^{1}H-NMR of Compound 26

Figure S56. ^{13}C-NMR of Compound 26

Mass Spectrum SmartFormula Report

Analysis Info

Analysis Name	D:\Data\20180410CEYANG\XY-N-6_1-C,8_01_11803.d	Acquisition Date	4/10/2018 12:41:24 PM
Method	20131026_ceyang b-ch3oh.m	Operator	Bruker Customer
Sample Name	XY-N-6	Instrument / Ser#	micrOTOF-Q 125
Comment			

Acquisition Parameter

Source Type	ESI	Ion Polarity	Positive	Set Nebulizer	1.2 Bar
Focus	Active	Set Capillary	4500 V	Set Dry Heater	180 °C
Scan Begin	50 m/z	Set End Plate Offset	-500 V	Set Dry Gas	8.0 l/min
Scan End	1500 m/z	Set Collision Cell RF	400.0 Vpp	Set Divert Valve	Source

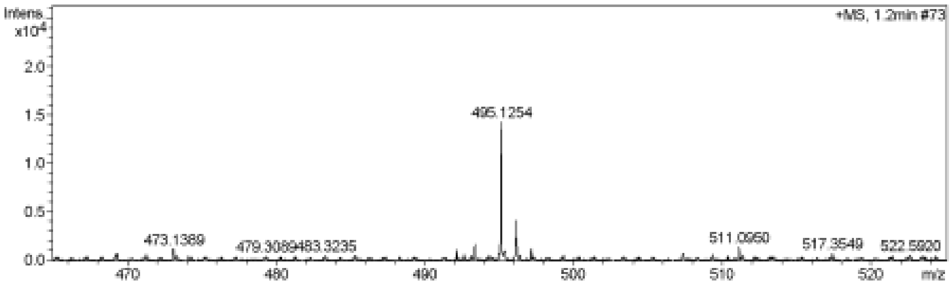

Meas. m/z	#	Formula	m/z	err [ppm]	Mean err [ppm]	rdb	N-Rule	e⁻ Conf	mSigma	Std I	Std Mean m/z	Std I VarNorm	Std m/z Diff	Std Comb Dev
495.1254														
	1	C 24 H 24 Na O 10	495.1262	1.6	1.5	12.5	ok	even	21.35	0.0443	0.0023	0.0161	0.0006	0.8427

Figure S1. HR-ESI-MS of Compound 1

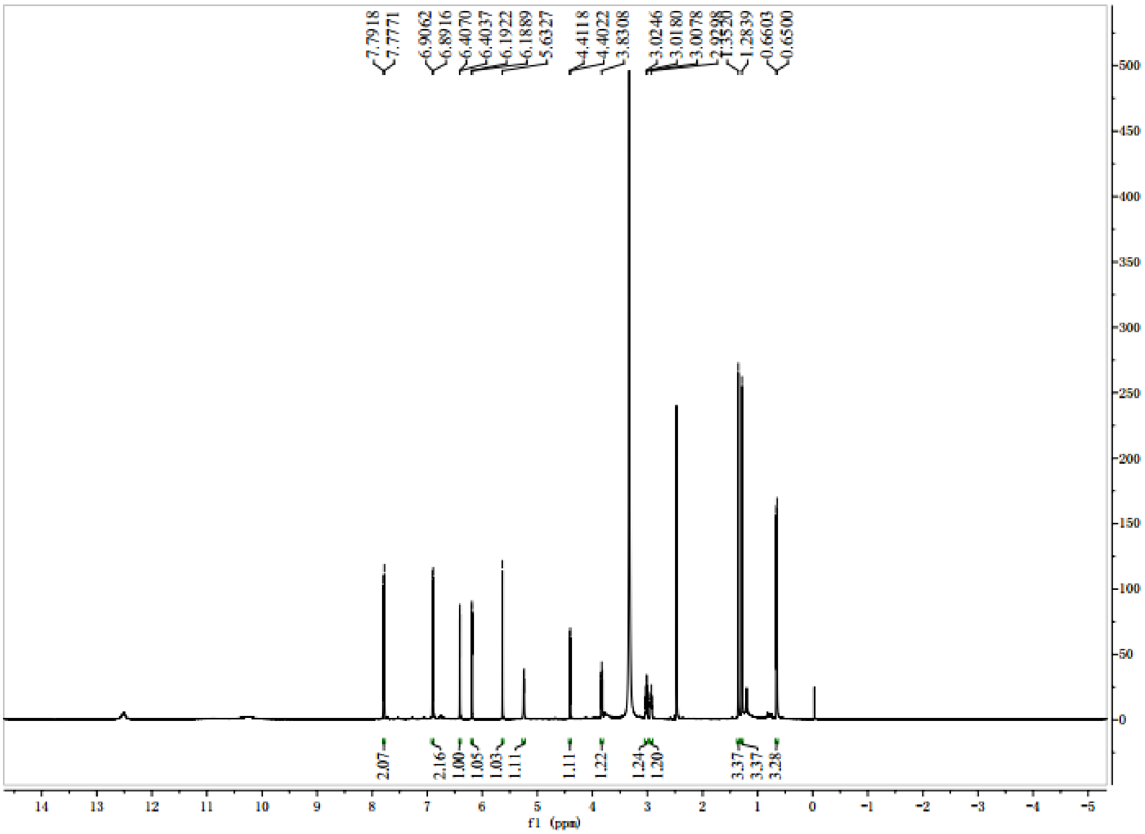

Figure S2. ^{1}H-NMR of Compound 1 (600MHz, DMSO-d_6)

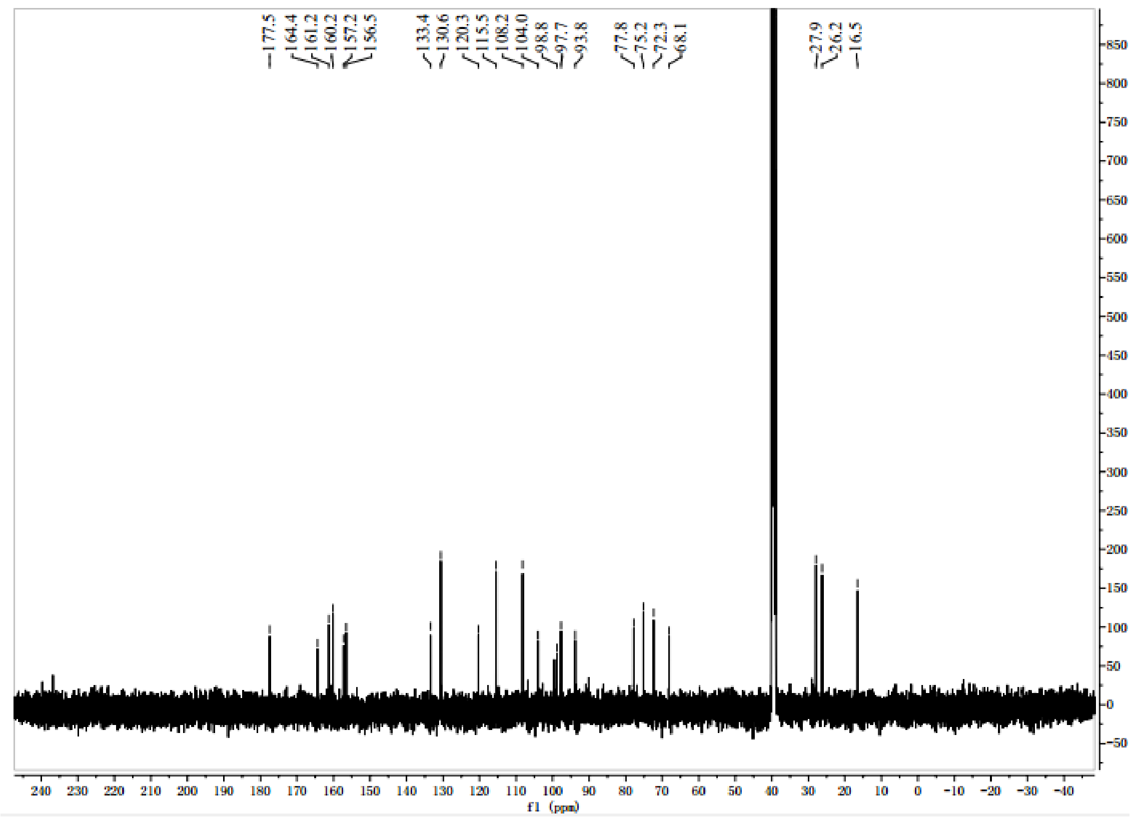

Figure S3. ^{13}C-NMR of Compound 1 (100MHz, DMSO-d_6)

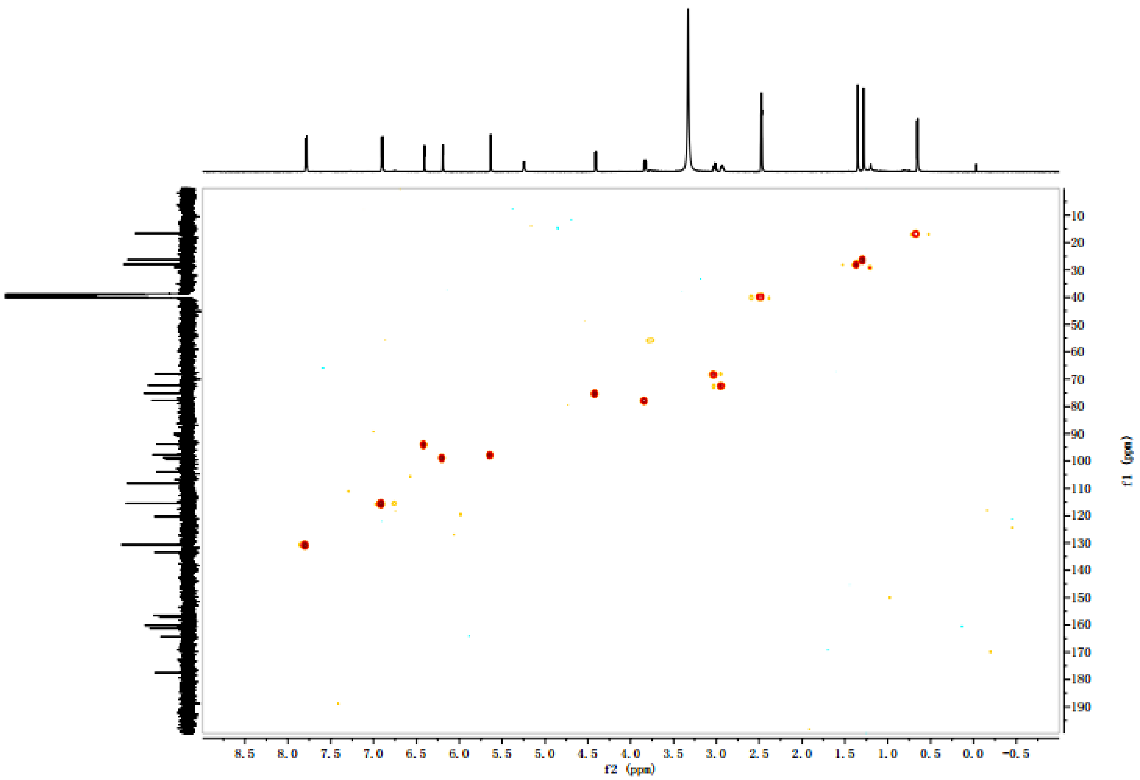

Figure S4. HSQC of Compound 1

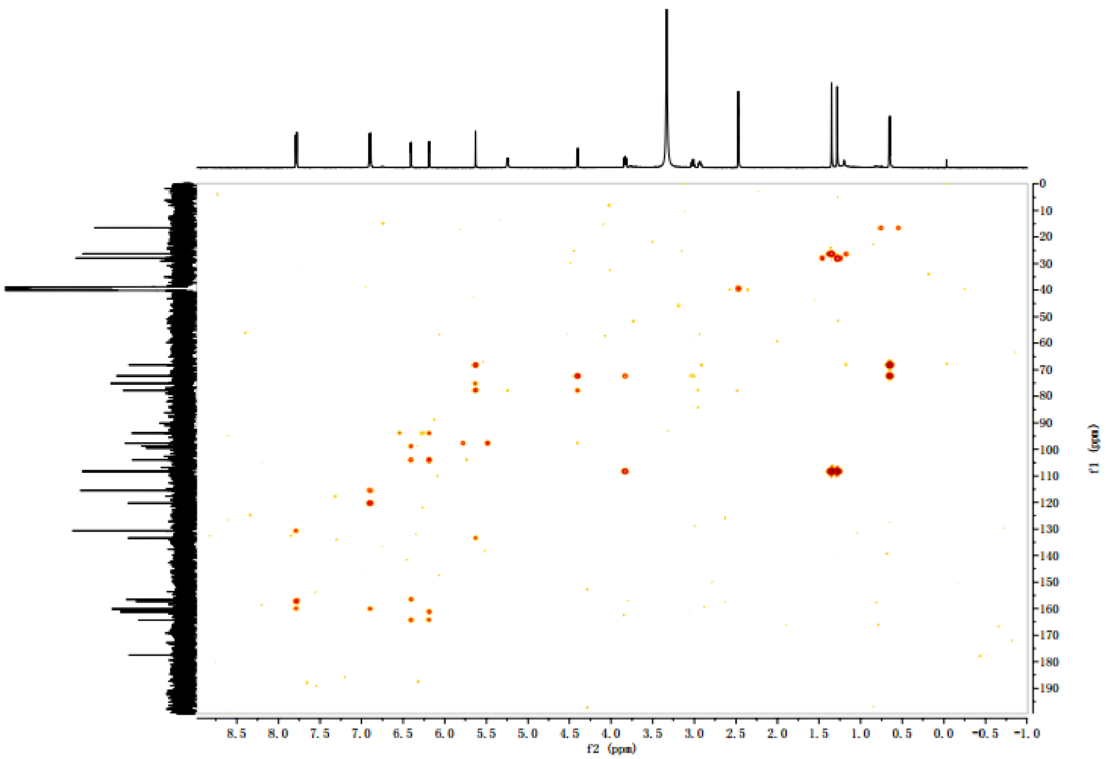

Figure S5. HMBC of Compound 1

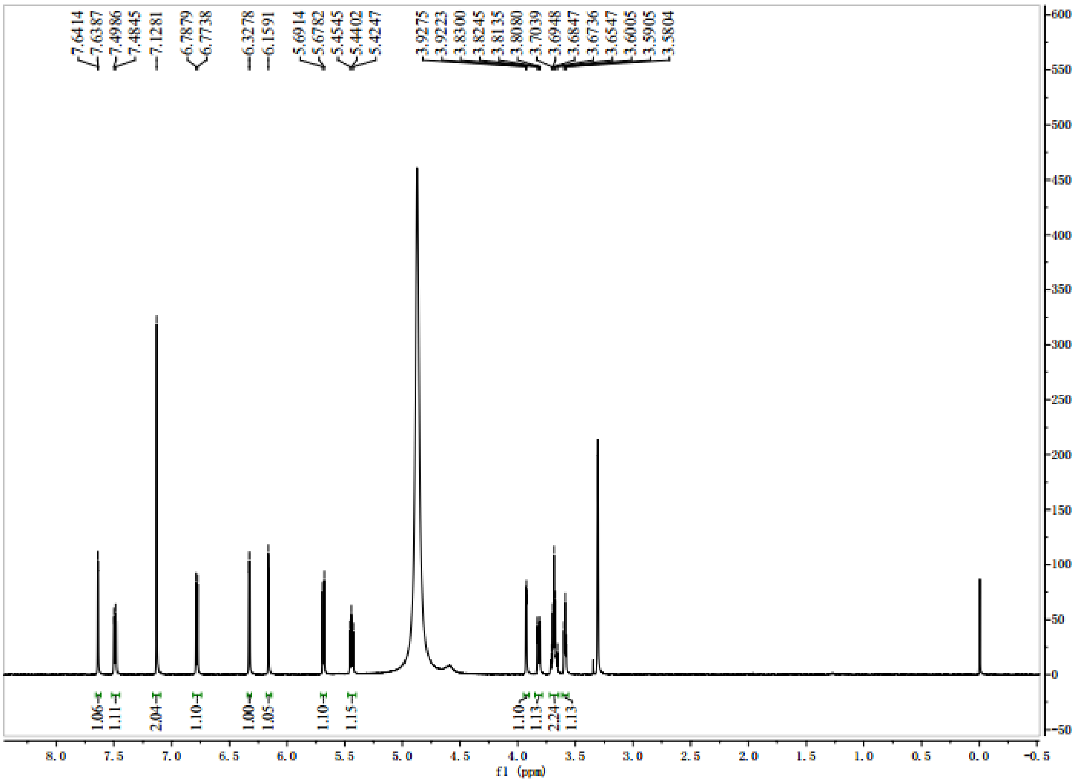

Figure S6. ^{1}H-NMR of Compound 2 (600MHz, CD_3OD)

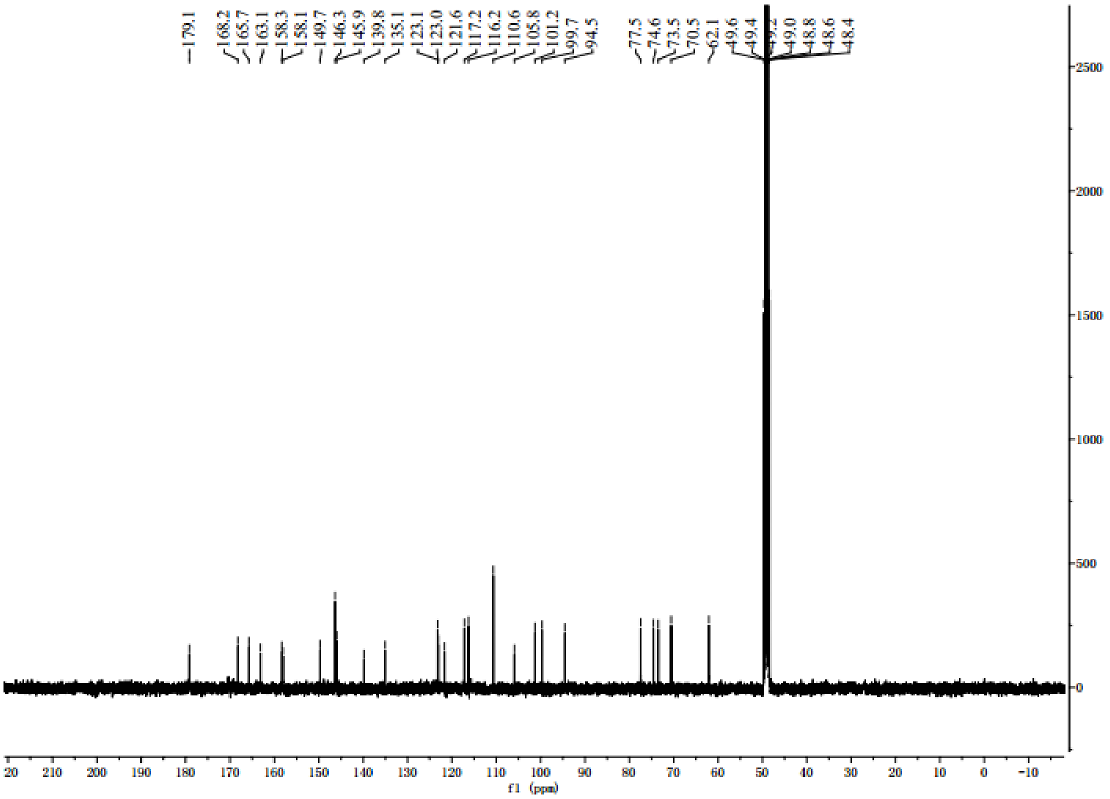

Figure S7. ^{13}C-NMR of Compound 2 (100MHz, CD_3OD)

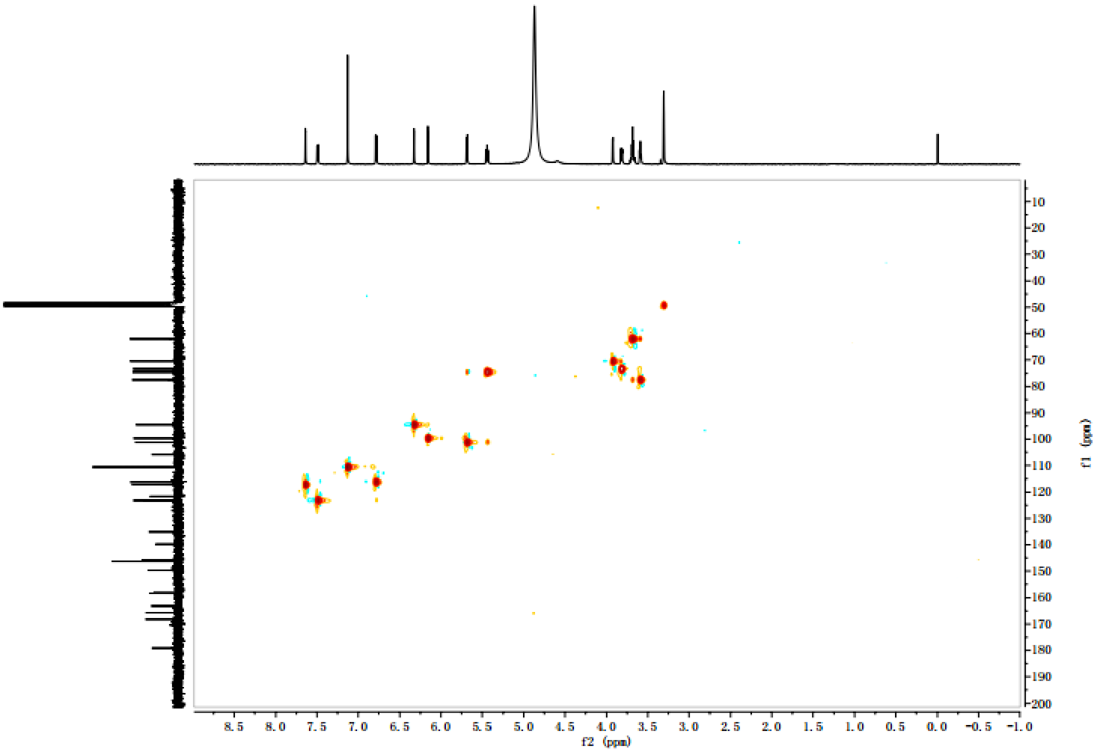

Figure S8. HSQC of Compound 2

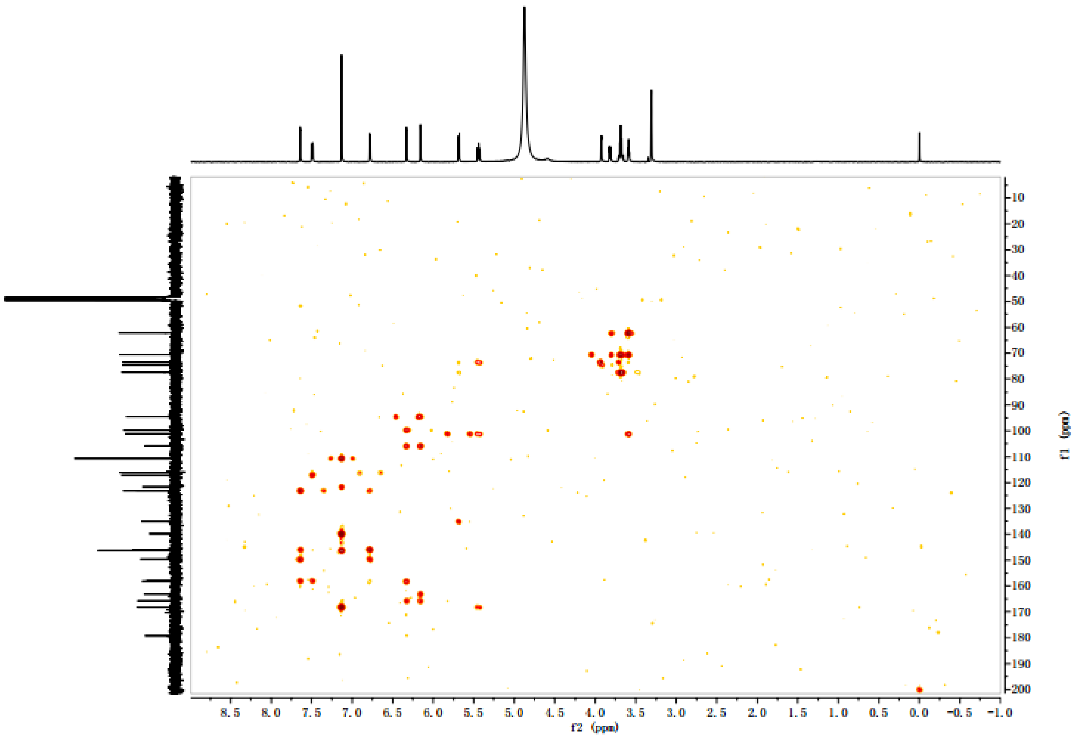

Figure S9. HMBC of Compound 2

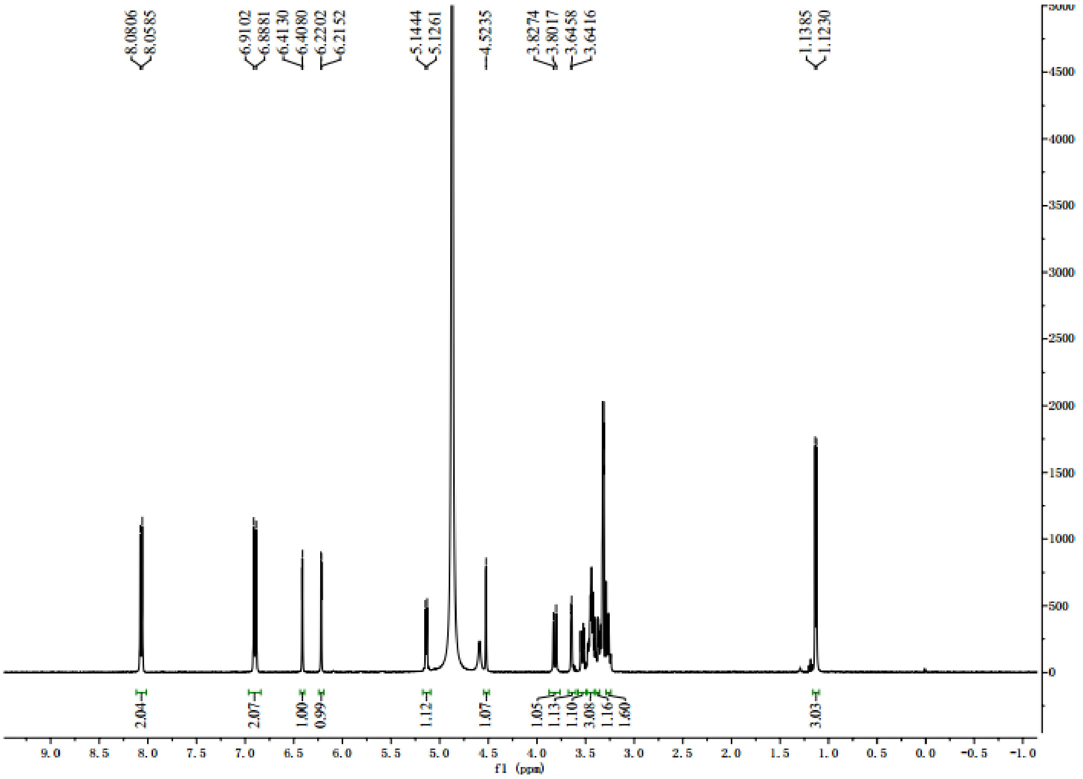

Figure S10. ^{1}H-NMR of Compound 3 (400MHz, CD_3OD)

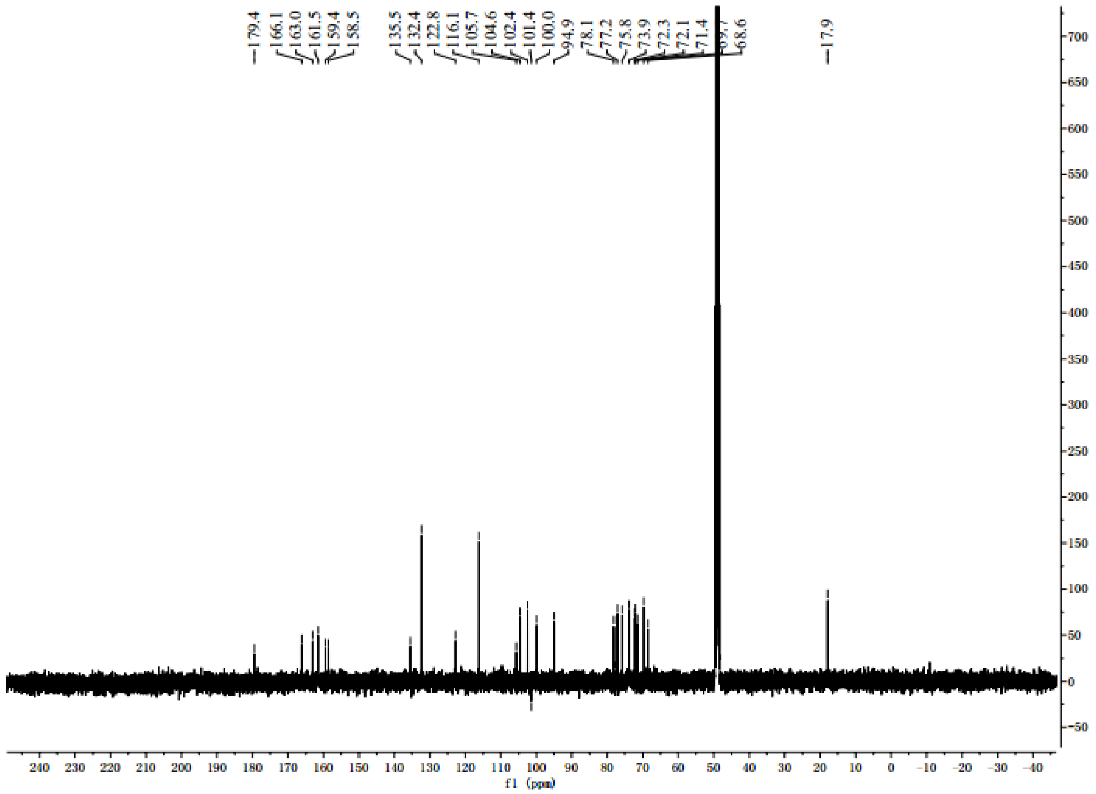

Figure S11. ^{13}C-NMR of Compound 3 (100MHz, CD_3OD)

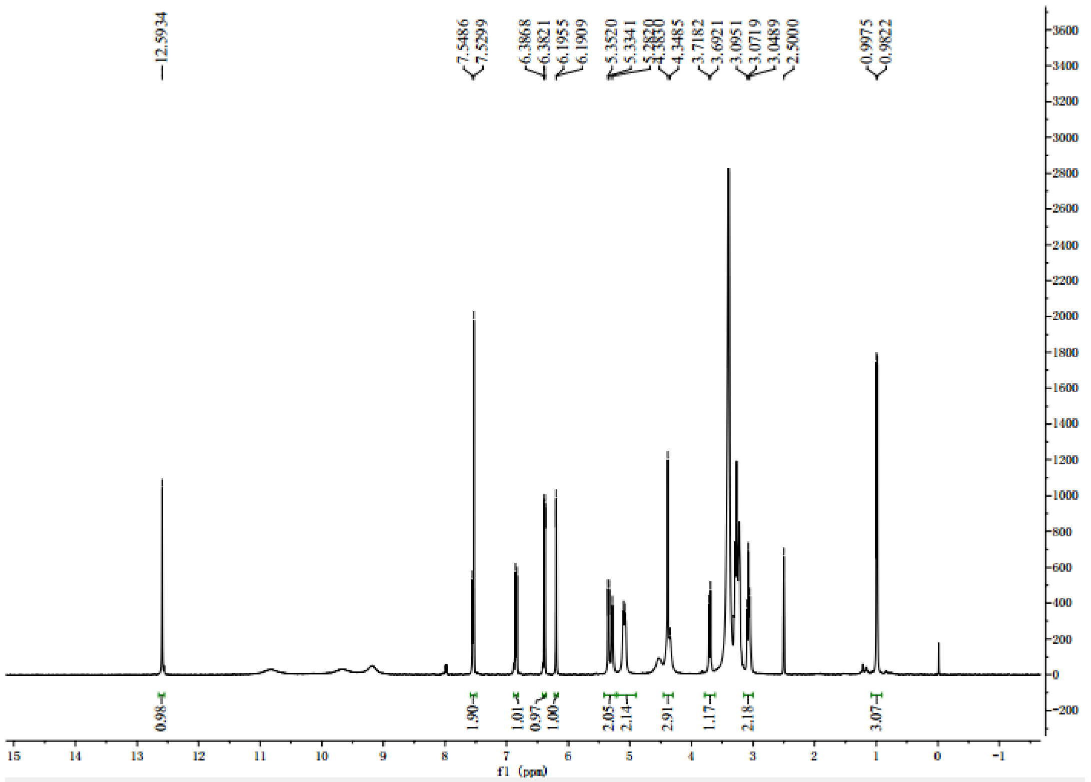

Figure S12. ^{1}H-NMR of Compound 4 (400MHz, DMSO-d_6)

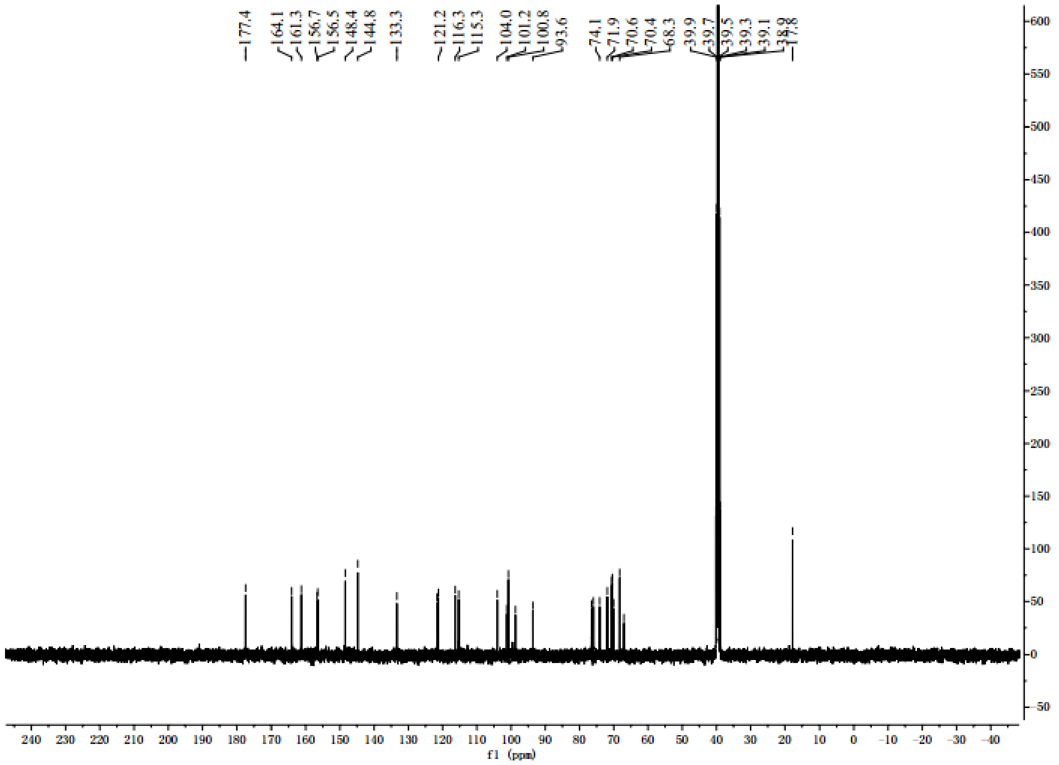

Figure S13. ^{13}C-NMR of Compound 4 (100MHz, DMSO-d_6)

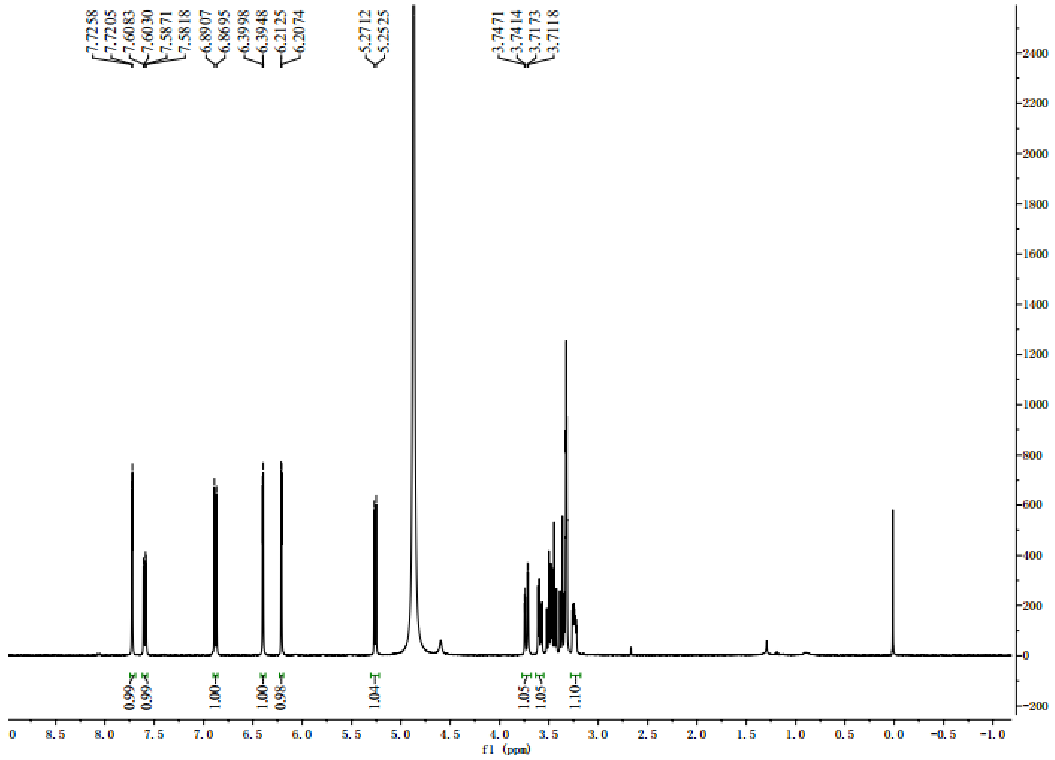

Figure S14. ^{1}H-NMR of Compound 5 (400MHz, CD_3OD)

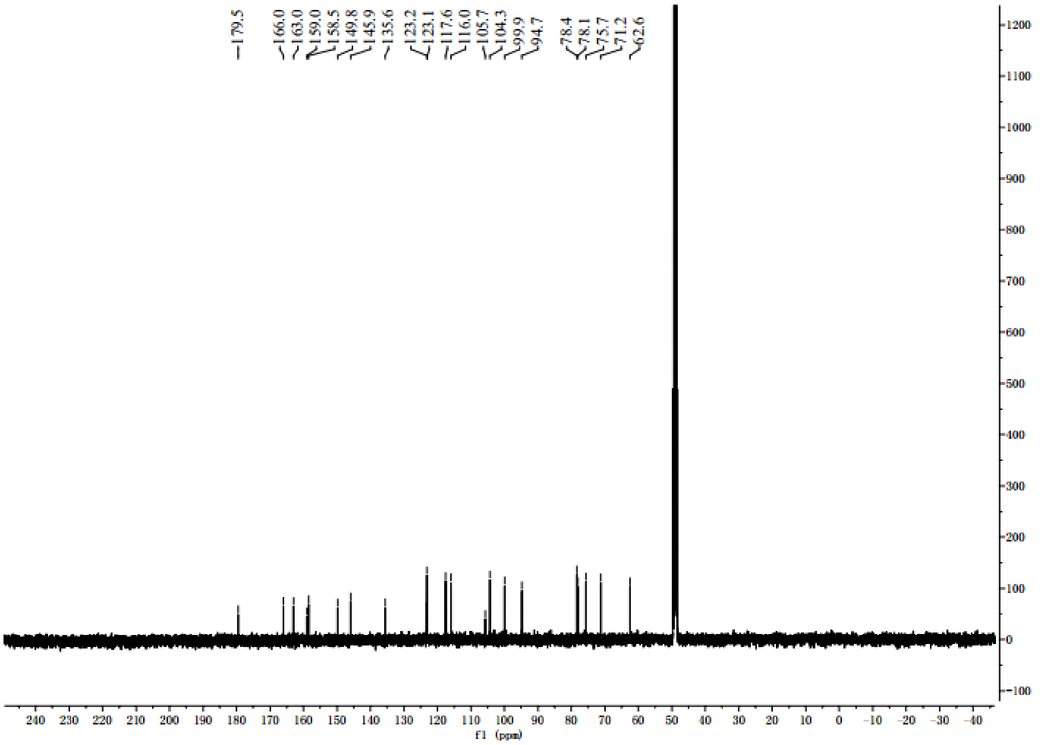

Figure S15. ^{13}C-NMR of Compound 5 (100MHz, CD_3OD)

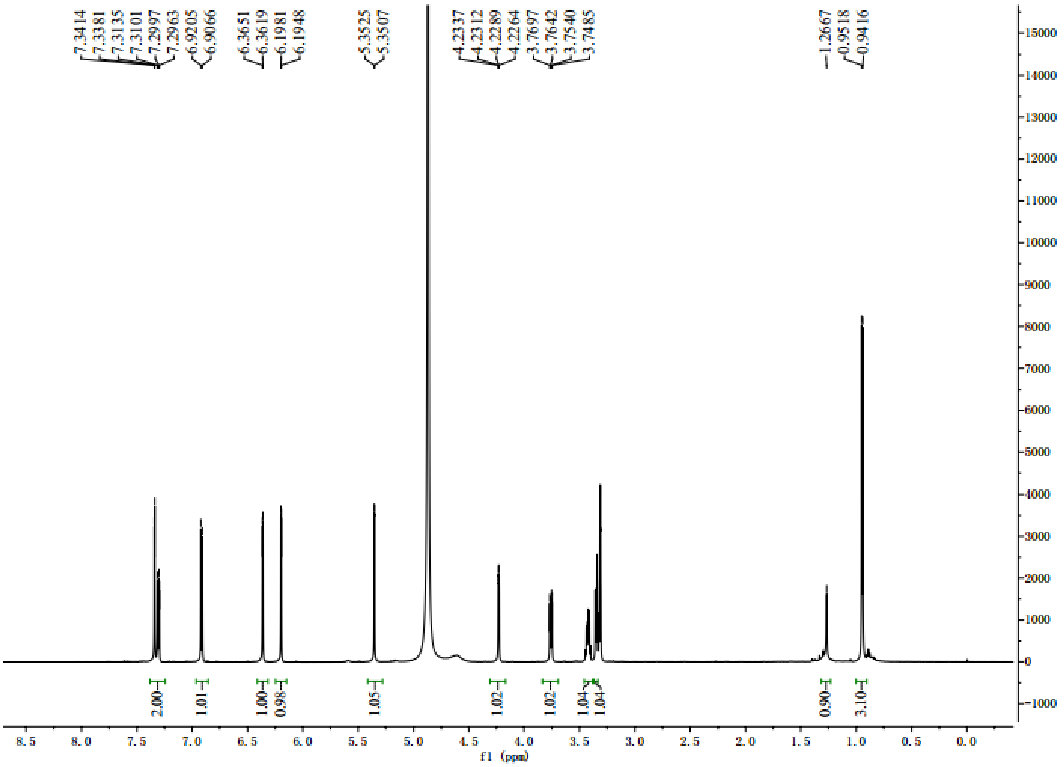

Figure S16. ^{1}H-NMR of Compound 6 (400MHz, CD_3OD)

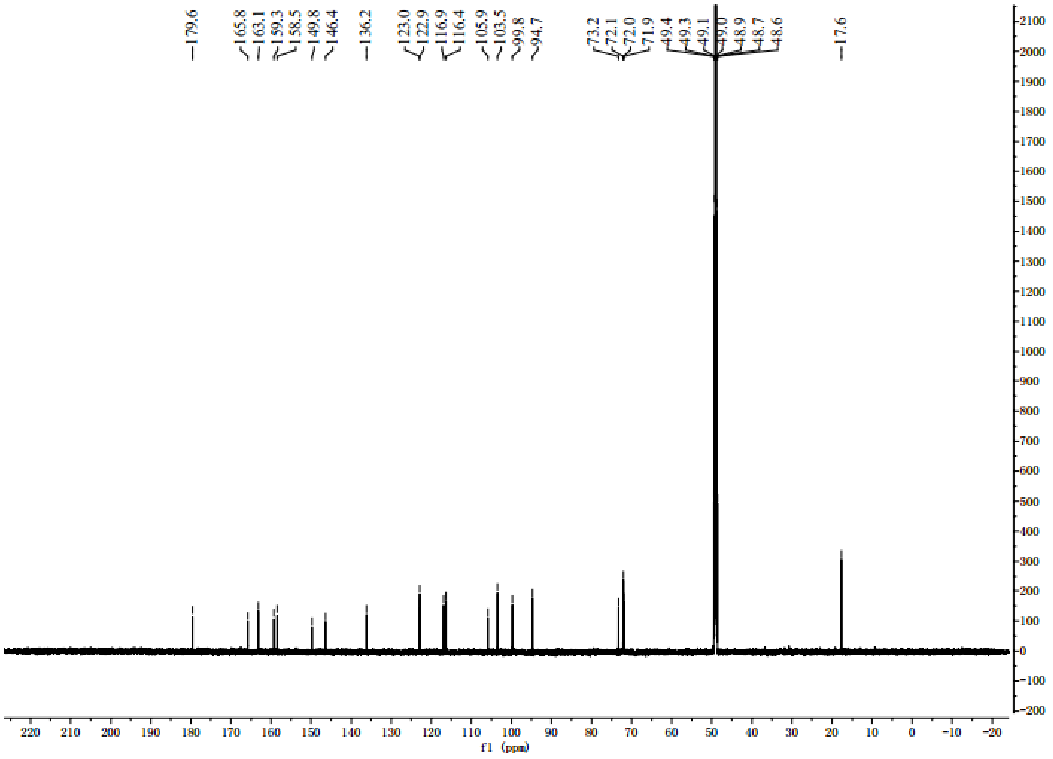

Figure S17. ^{13}C-NMR of Compound 6 (100MHz, CD_3OD)

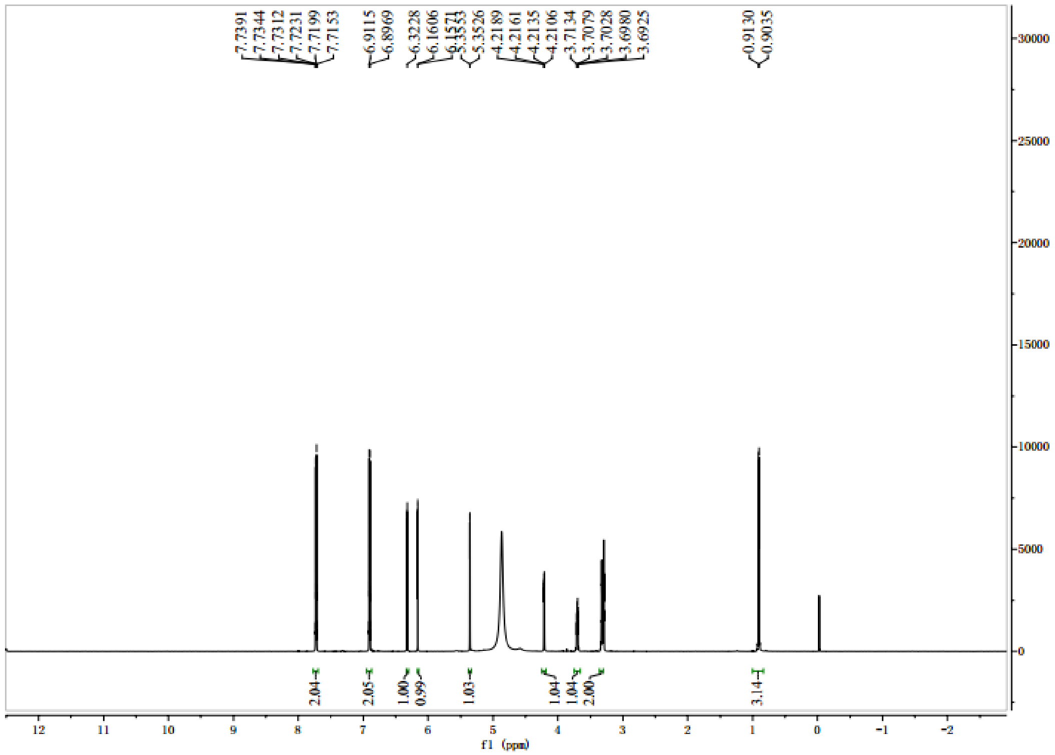

Figure S18. ^{1}H-NMR of Compound 7 (400MHz, CD_3OD)

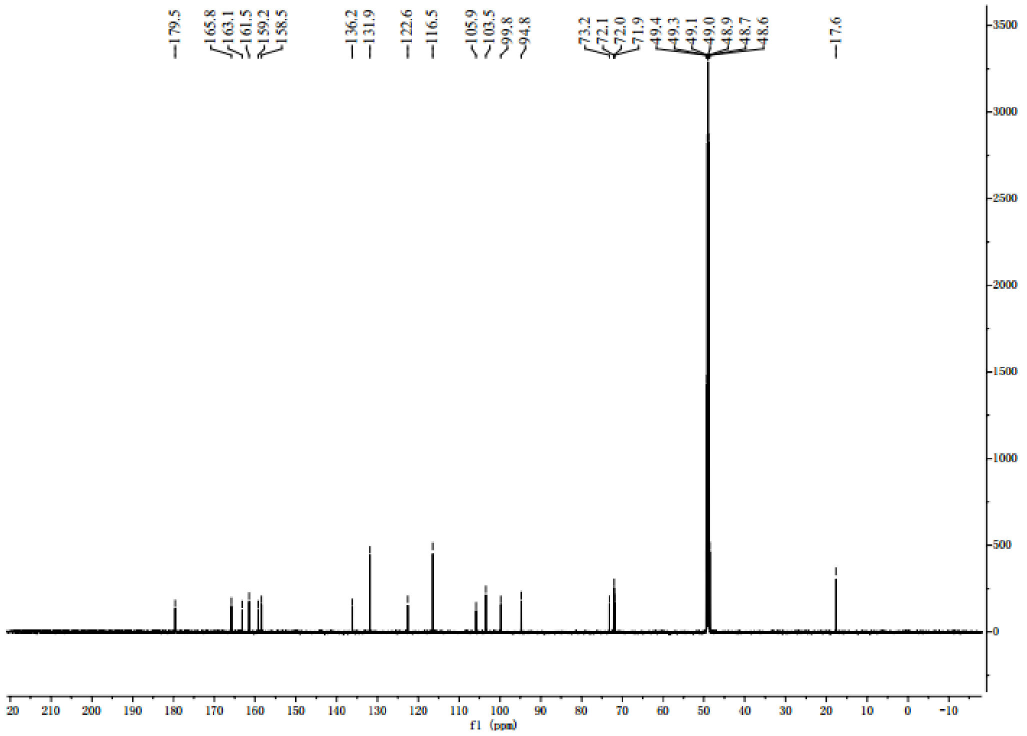

Figure S19. ^{13}C-NMR of Compound 7 (100MHz, CD_3OD)

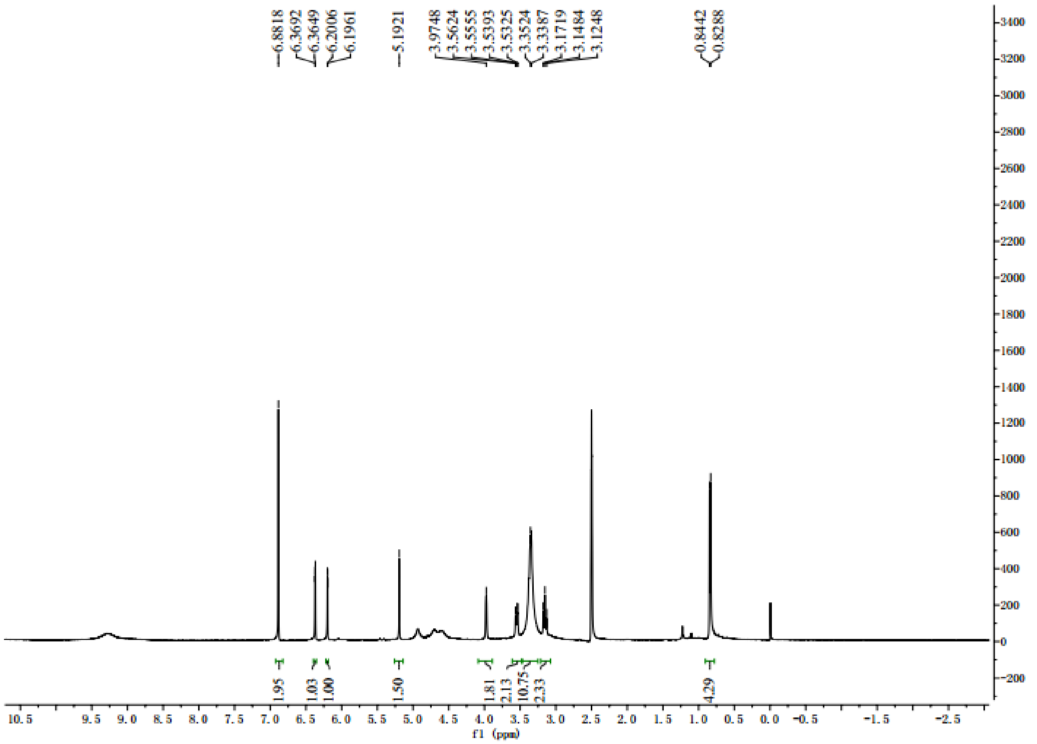

Figure S20. ^{1}H-NMR of Compound 8 (400MHz, DMSO-d_6)

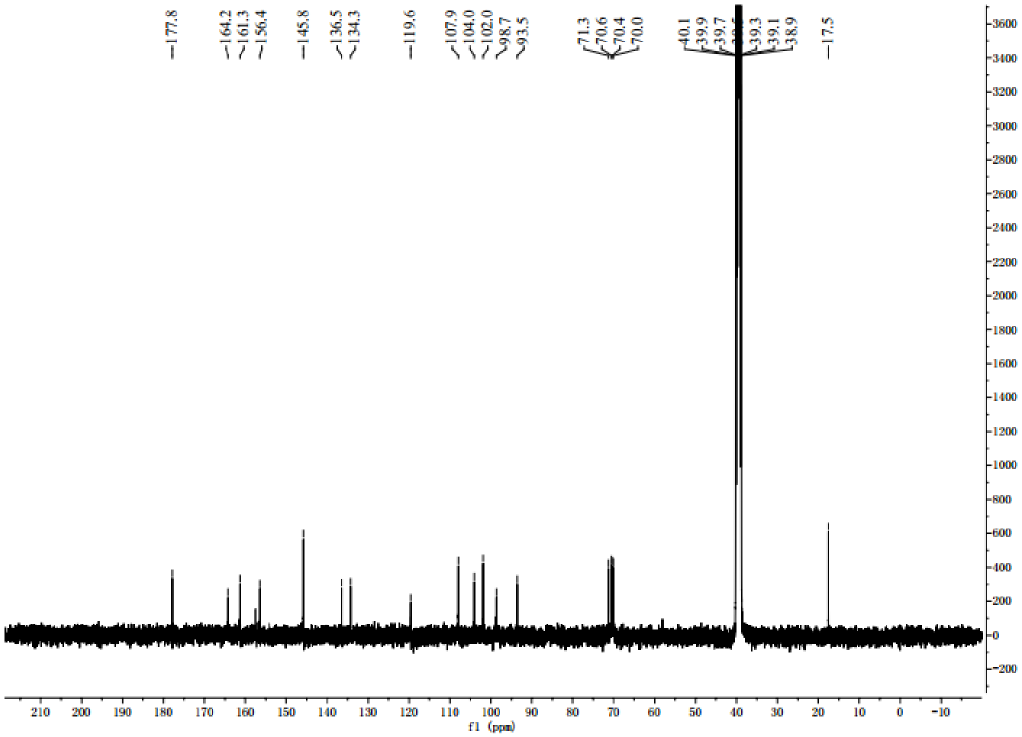

Figure S21. ^{13}C-NMR of Compound 8 (100MHz, DMSO-d_6)

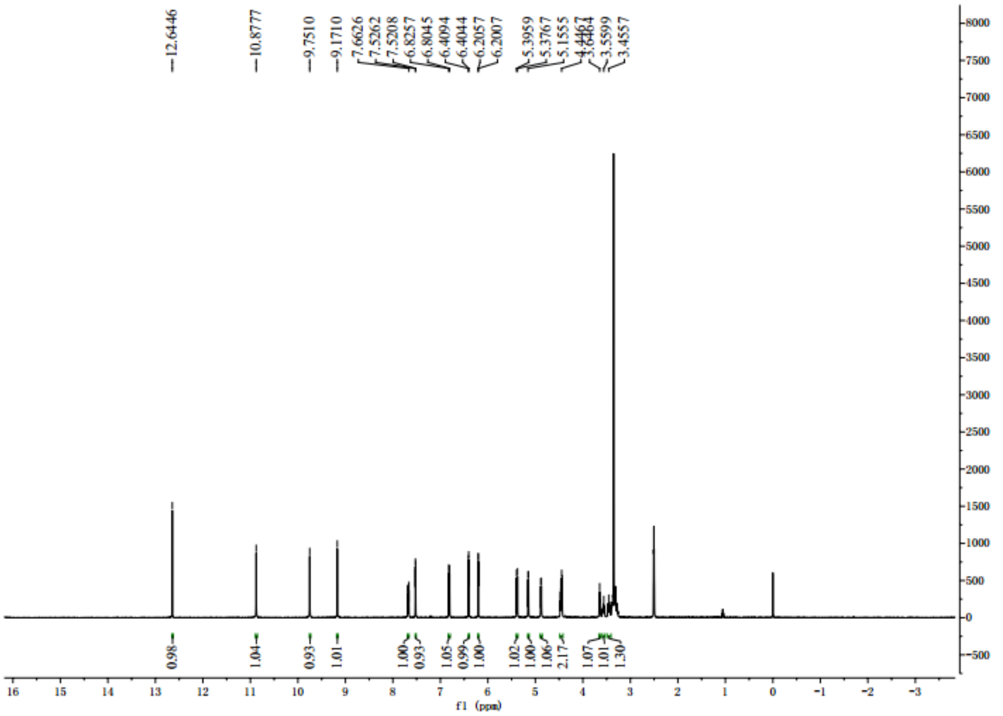

Figure S22. ^{1}H-NMR of Compound 9 (400MHz, DMSO-d_6)

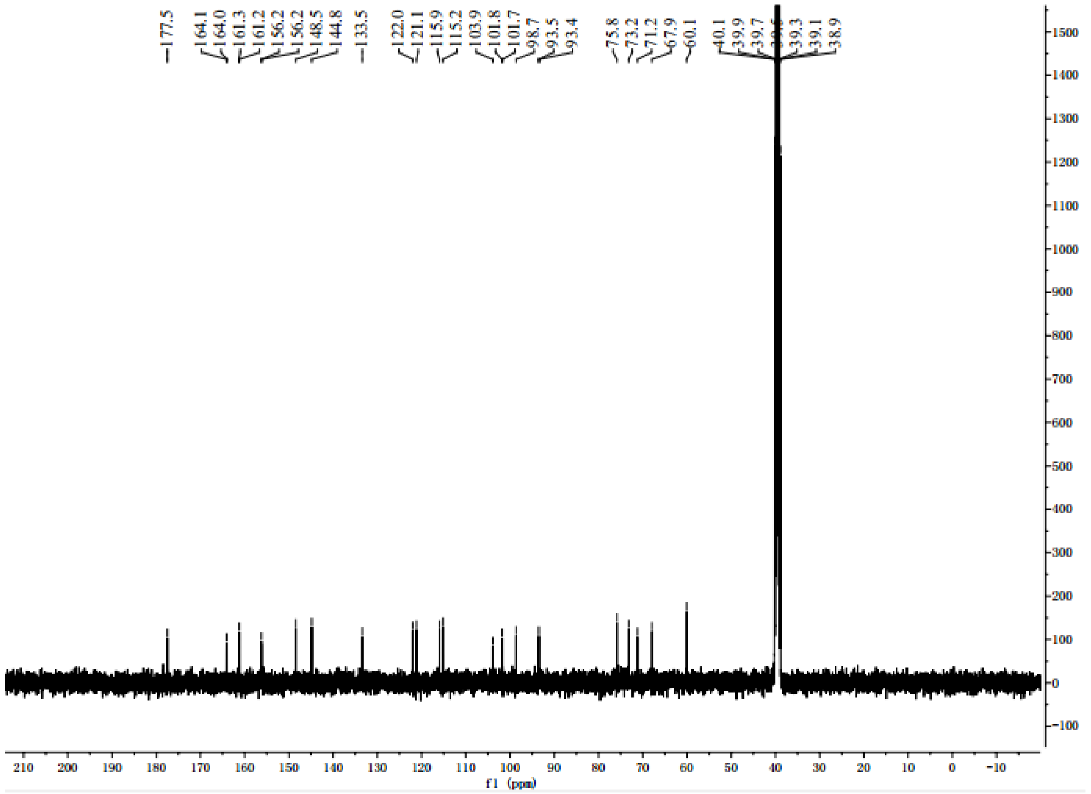

Figure S23. ^{13}C-NMR of Compound 9 (100MHz, DMSO-d_6)

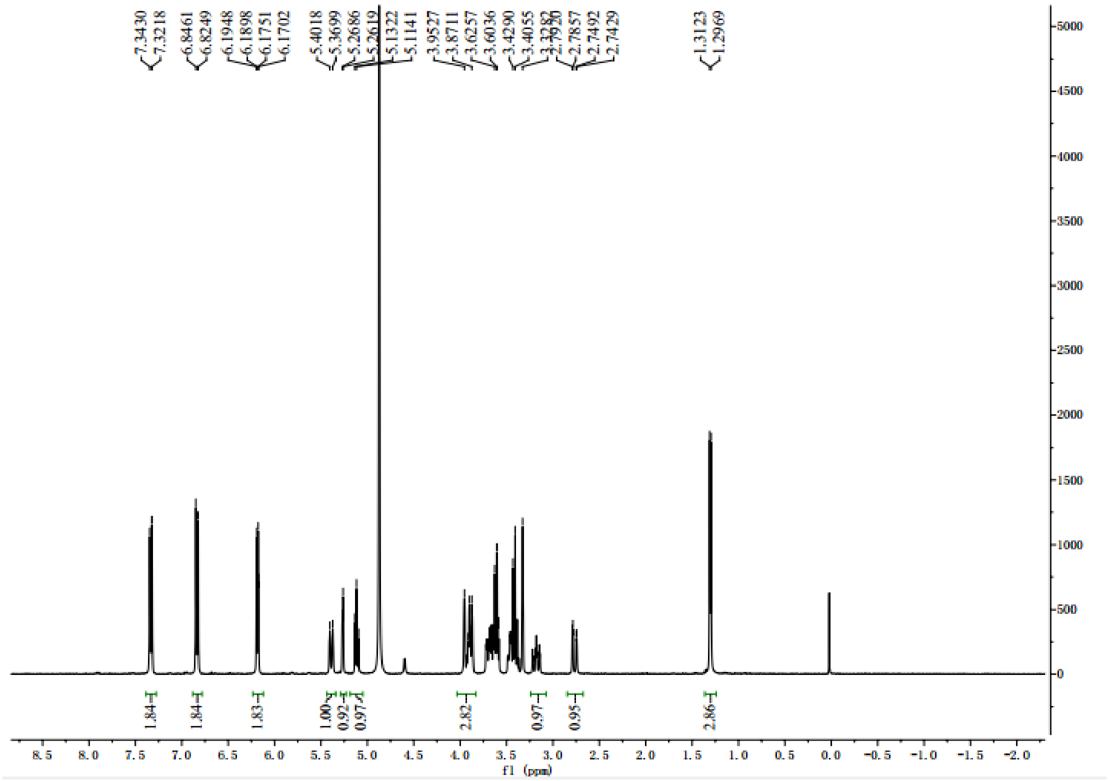

Figure S24. ^{1}H-NMR of Compound 10 (400MHz, CD_3OD)

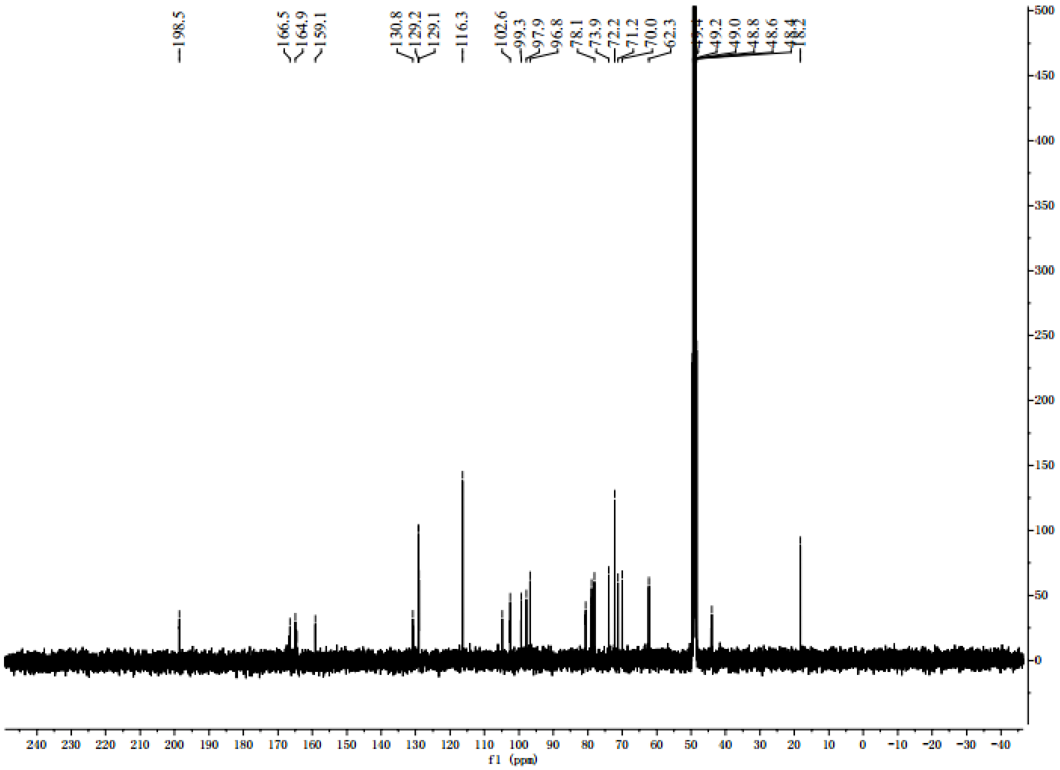

Figure S25. ^{13}C-NMR of Compound 10 (100MHz, CD_3OD)

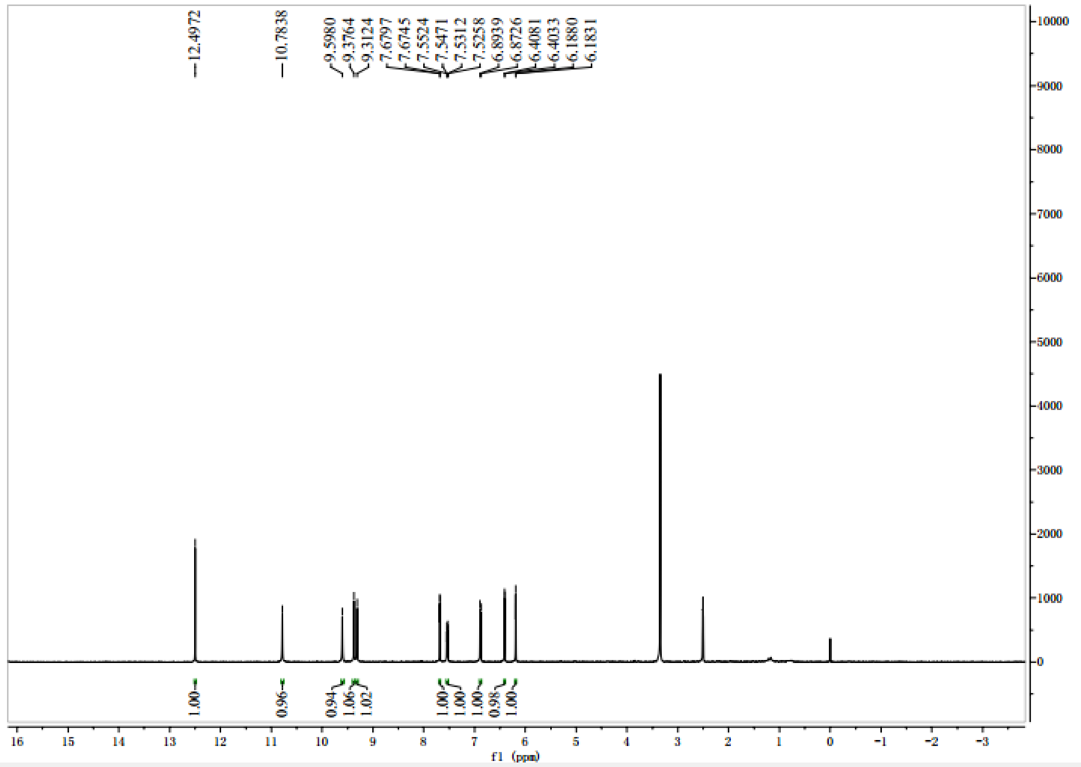

Figure S26. ^{1}H-NMR of Compound 11 (400MHz, DMSO-d_6)

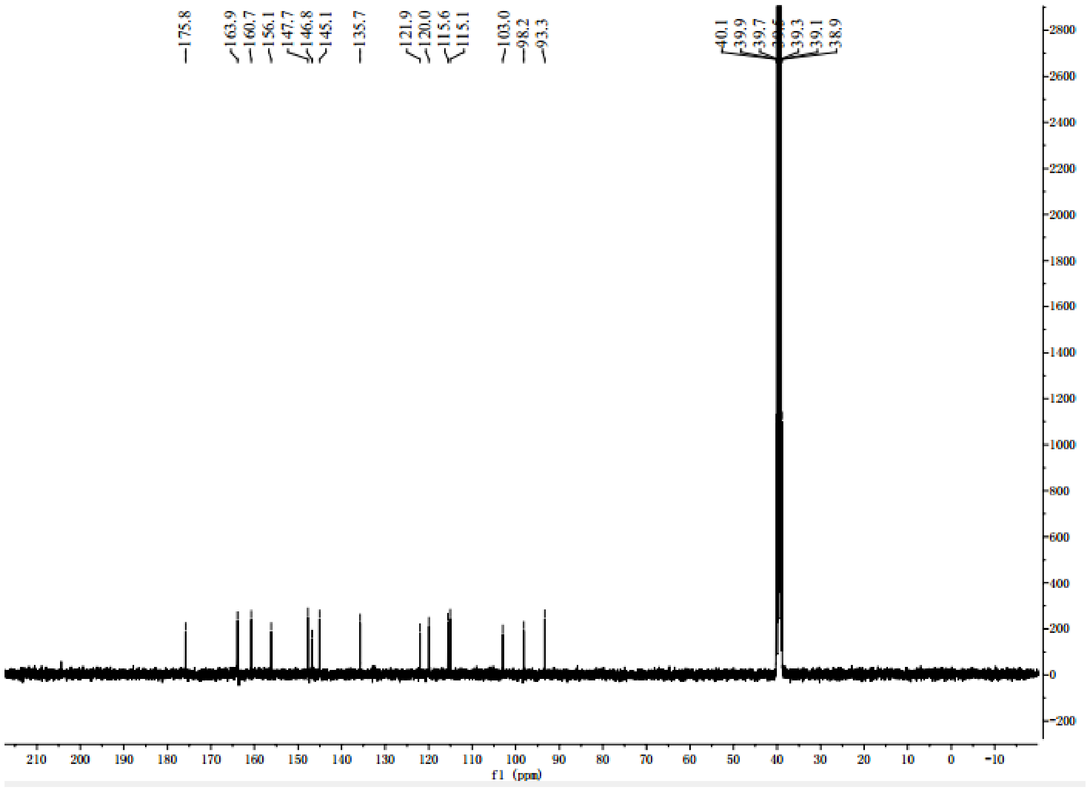

Figure S27. ^{13}C-NMR of Compound 11 (100MHz, DMSO-d_6)

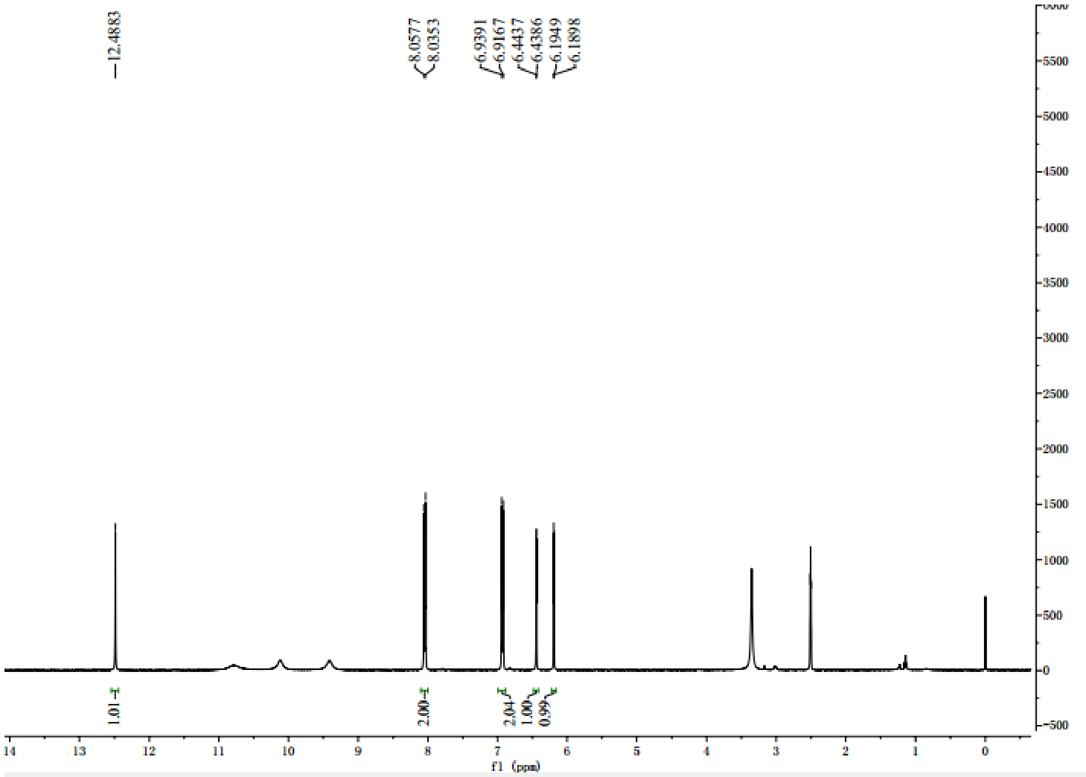

Figure S28. ^{1}H-NMR of Compound 12 (400MHz, DMSO-d_6)

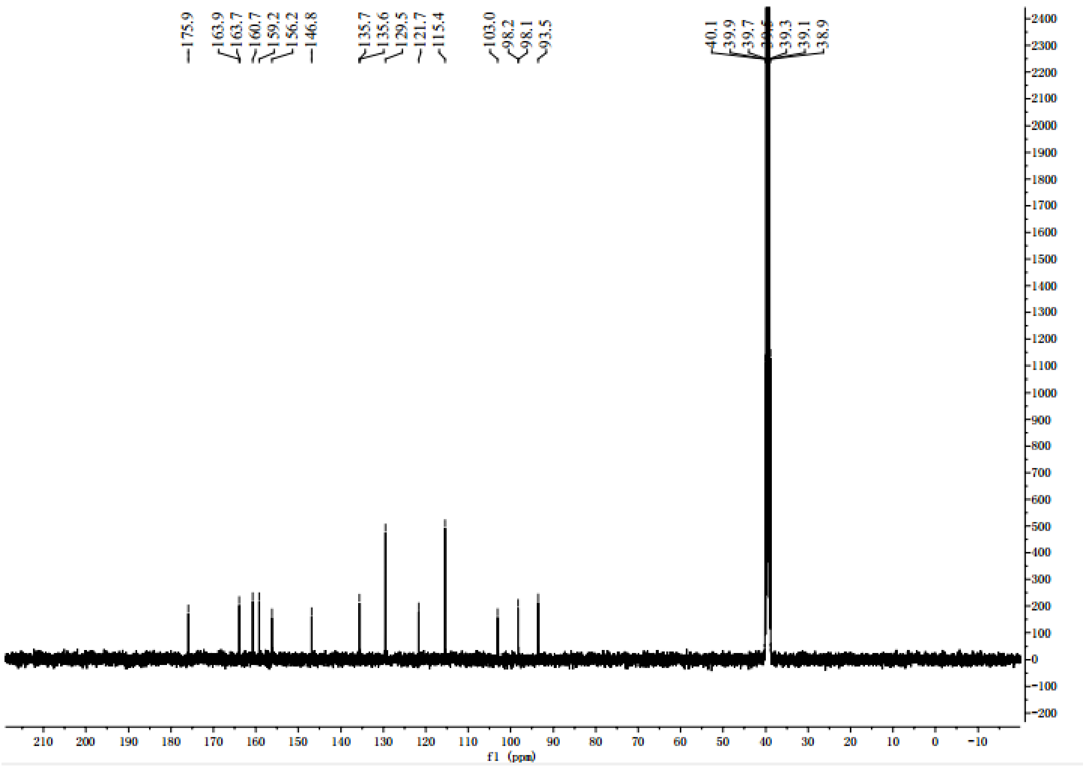

Figure S29. ^{13}C-NMR of Compound 12 (100MHz, DMSO-d_6)

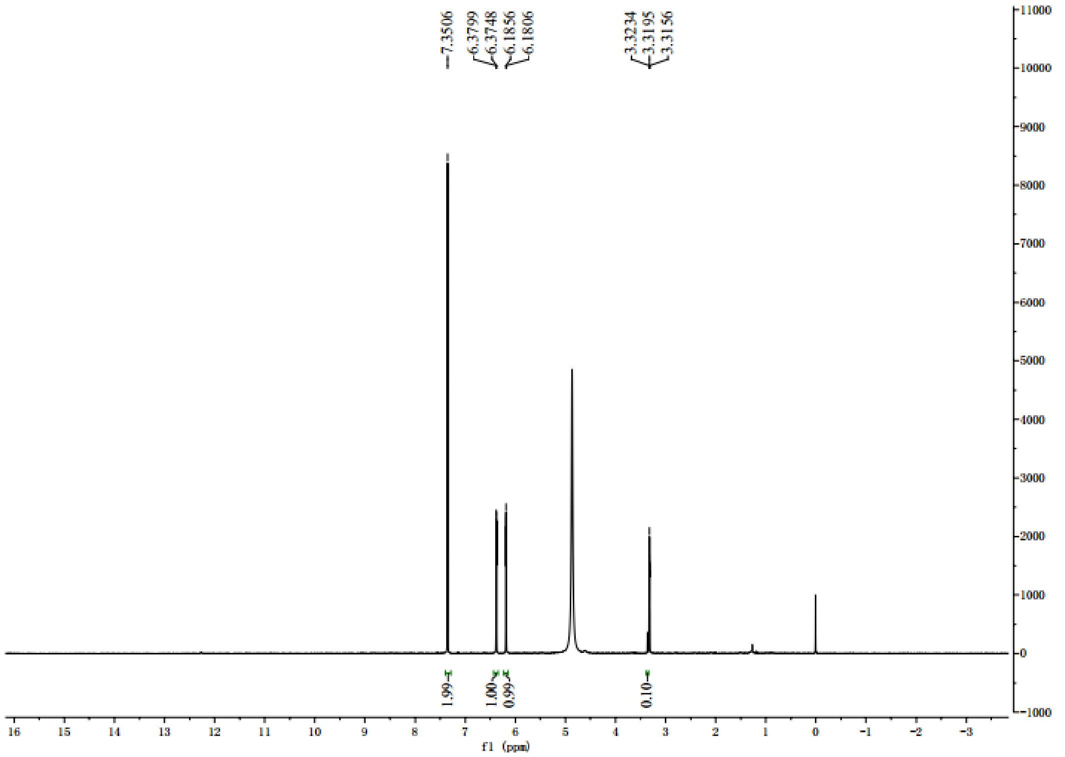

Figure S30. ^{1}H-NMR of Compound 13 (400MHz, CD_3OD)

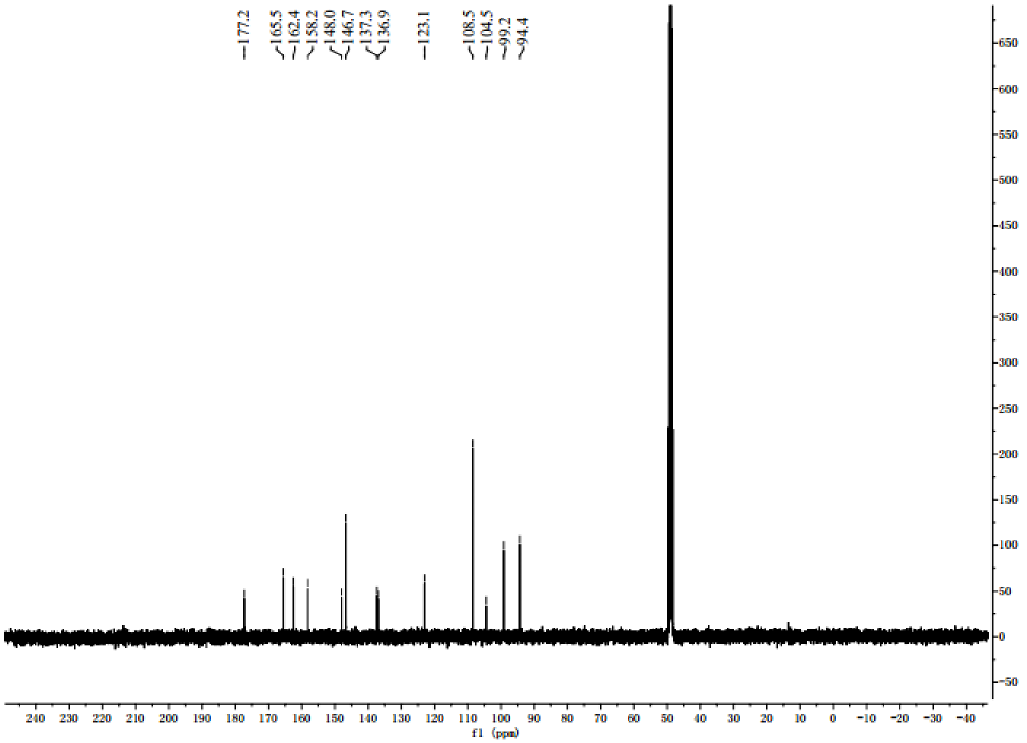

Figure S31. ^{13}C-NMR of Compound 13 (100MHz, CD_3OD)

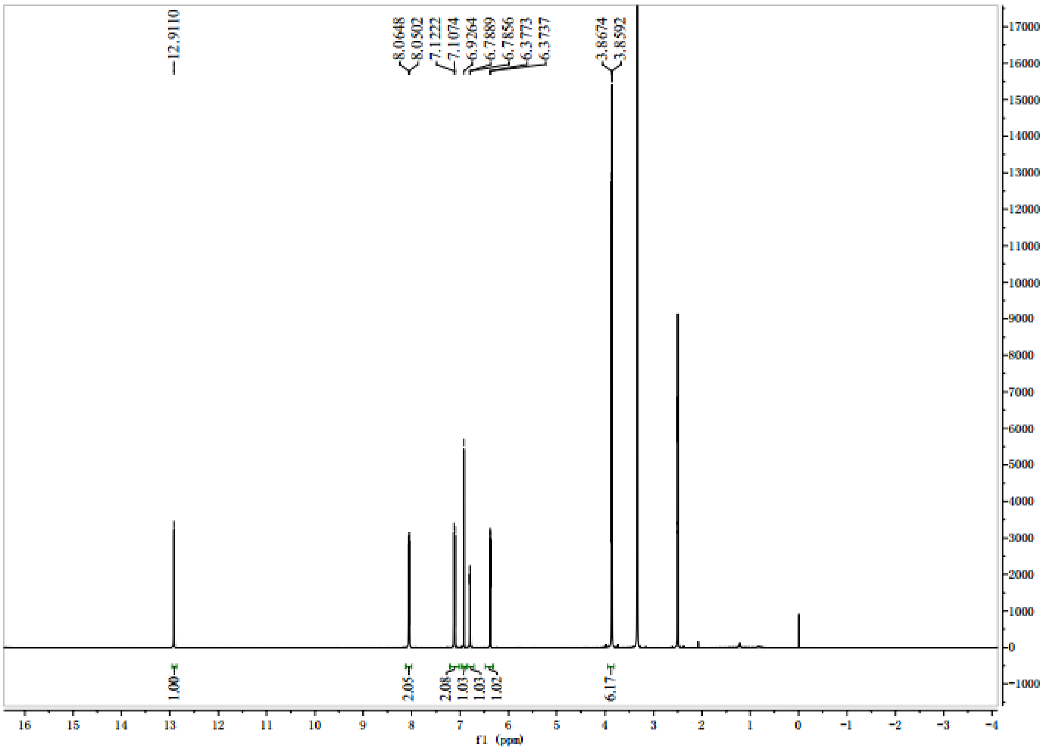

Figure S32. ^{1}H-NMR of Compound 14 (400MHz, DMSO-d_6)

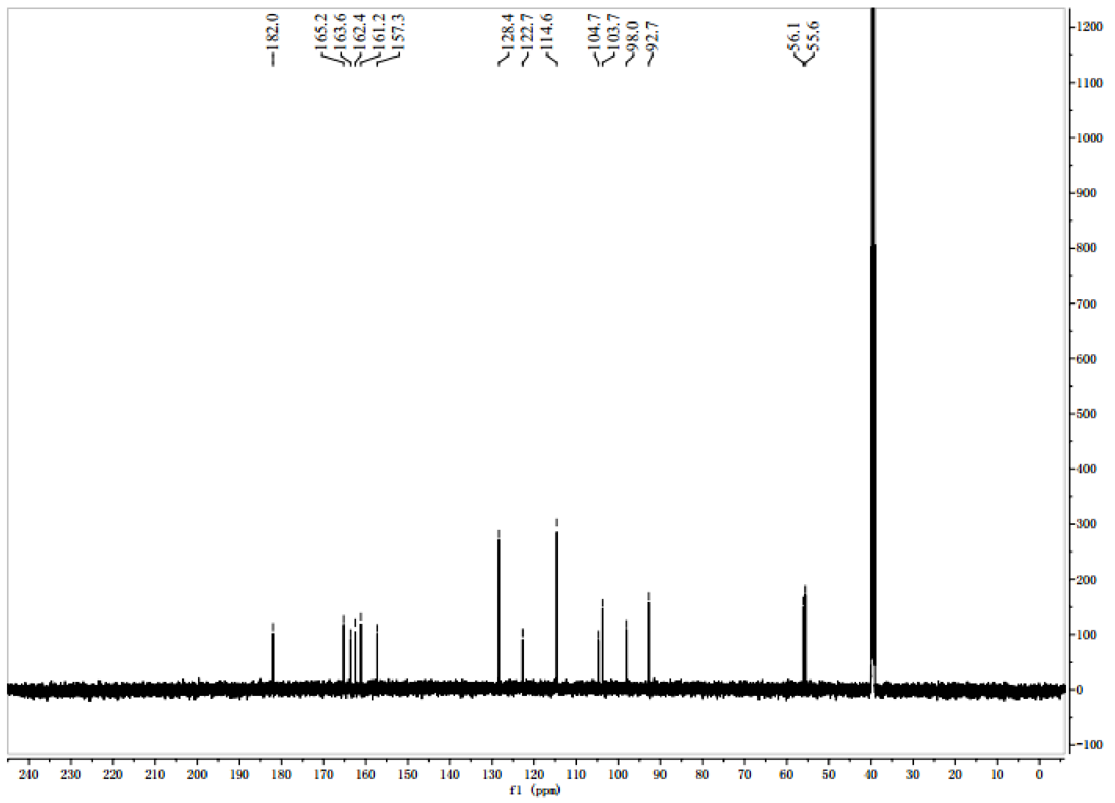

Figure S33. ^{13}C-NMR of Compound 14 (100MHz, DMSO-d_6)

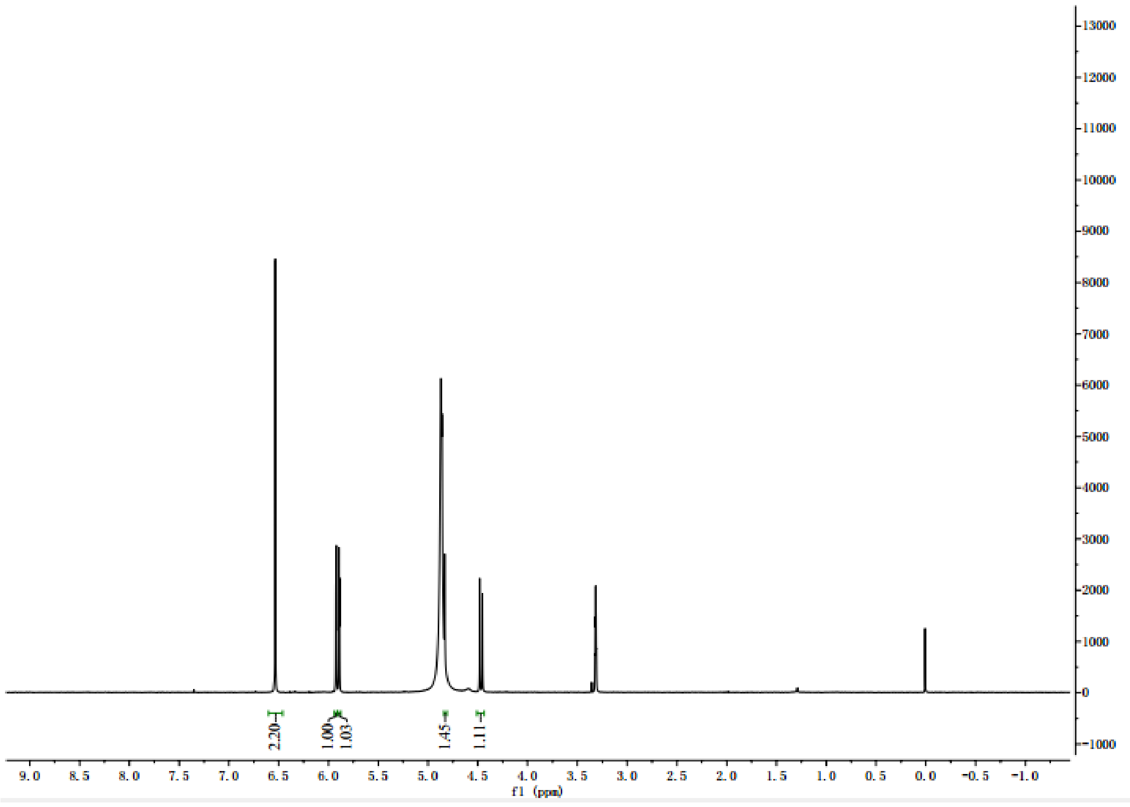

Figure S34. ^{1}H-NMR of Compound 15 (400MHz, CD_3OD)

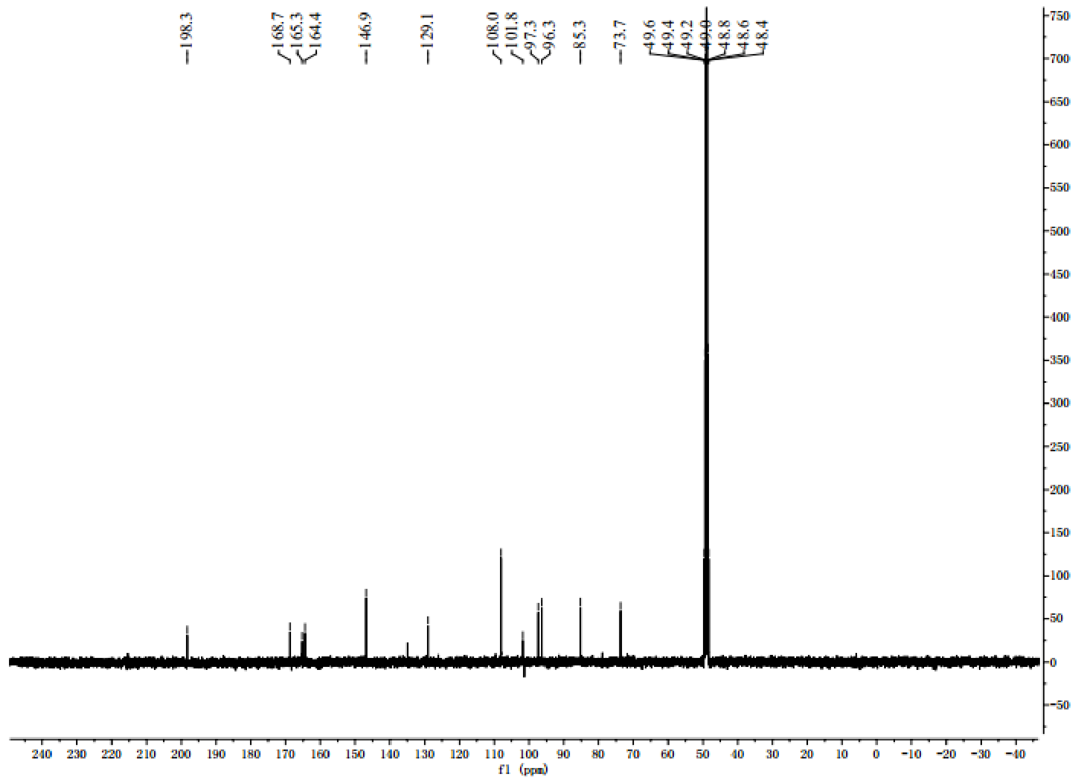

Figure S35. ^{13}C-NMR of Compound 15 (100MHz, CD_3OD)

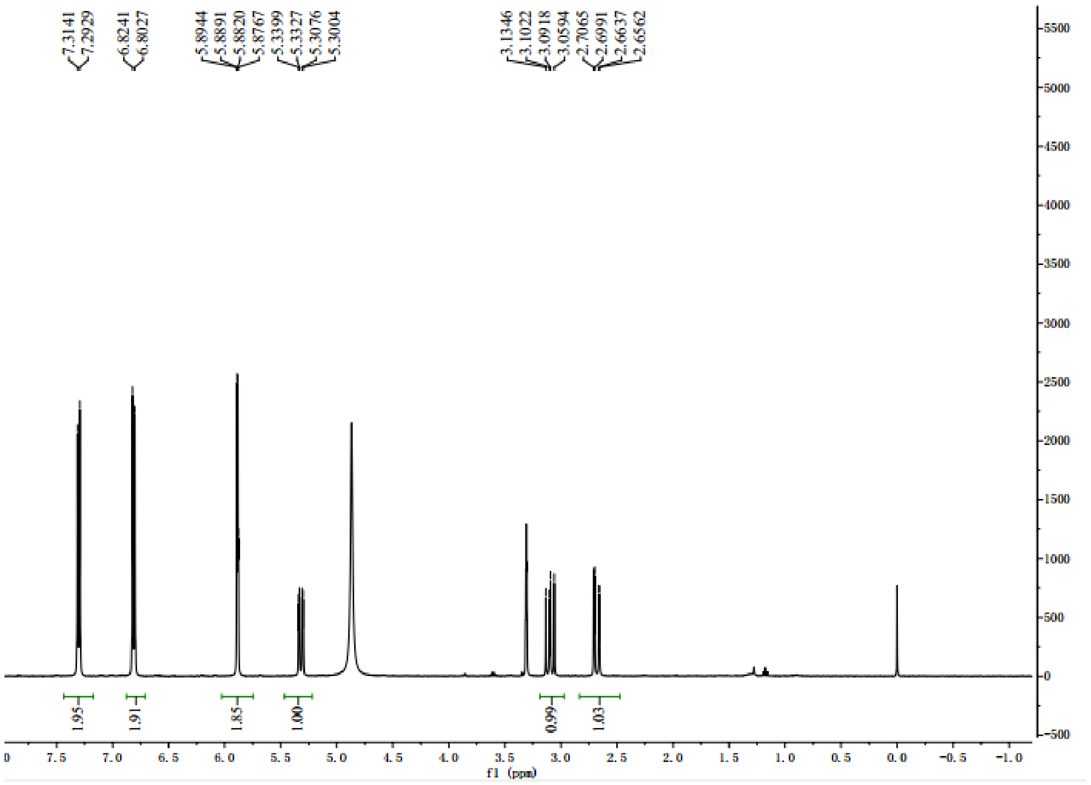

Figure S36. ^{1}H-NMR of Compound 16 (400MHz, CD_3OD)

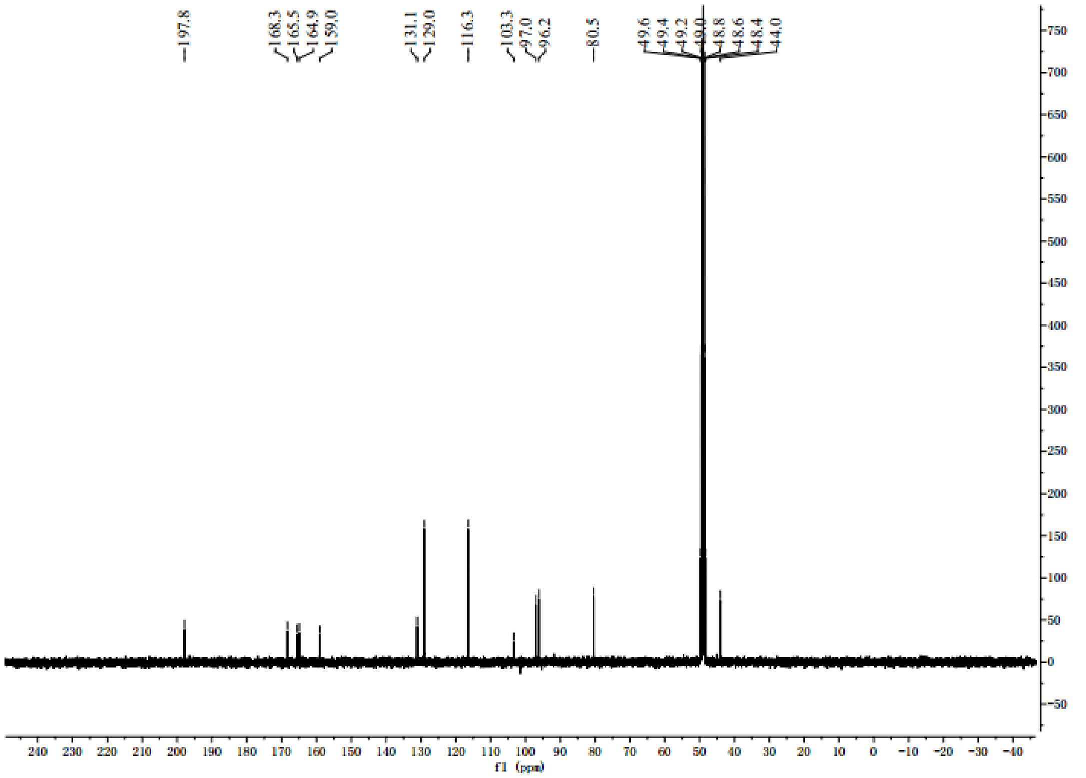

Figure S37. ^{13}C-NMR of Compound 16 (100MHz, CD_3OD)

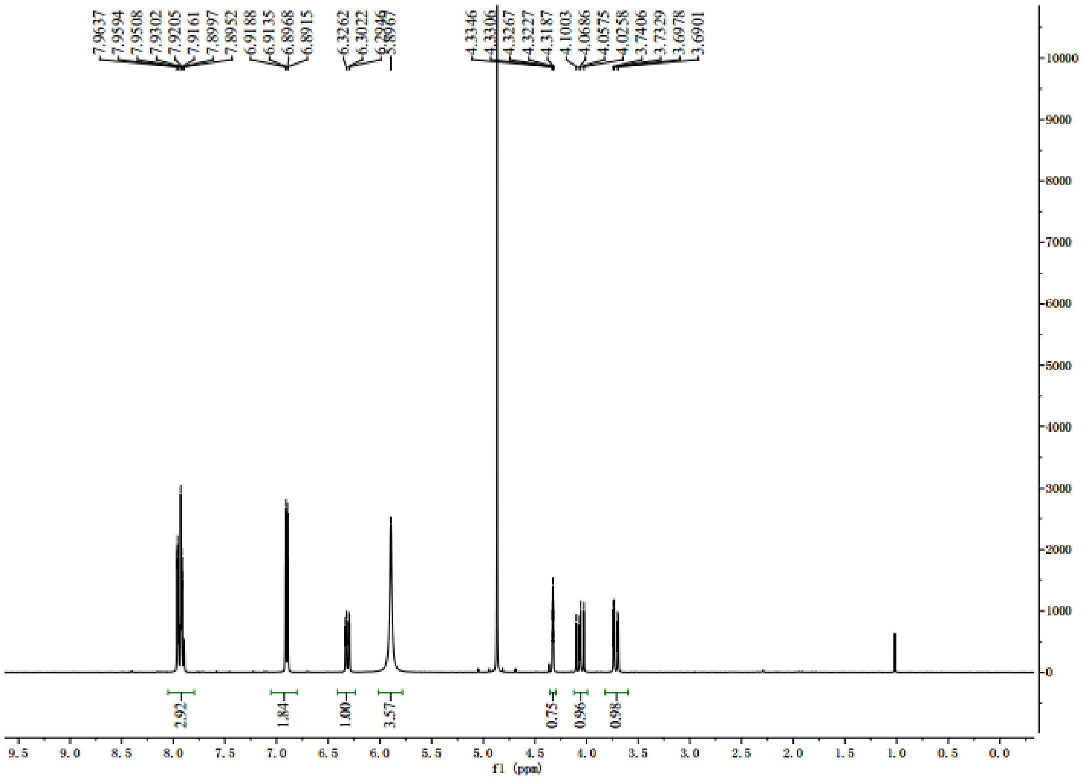

Figure S38. ^{1}H-NMR of Compound 17 (400MHz, CD_3OD)

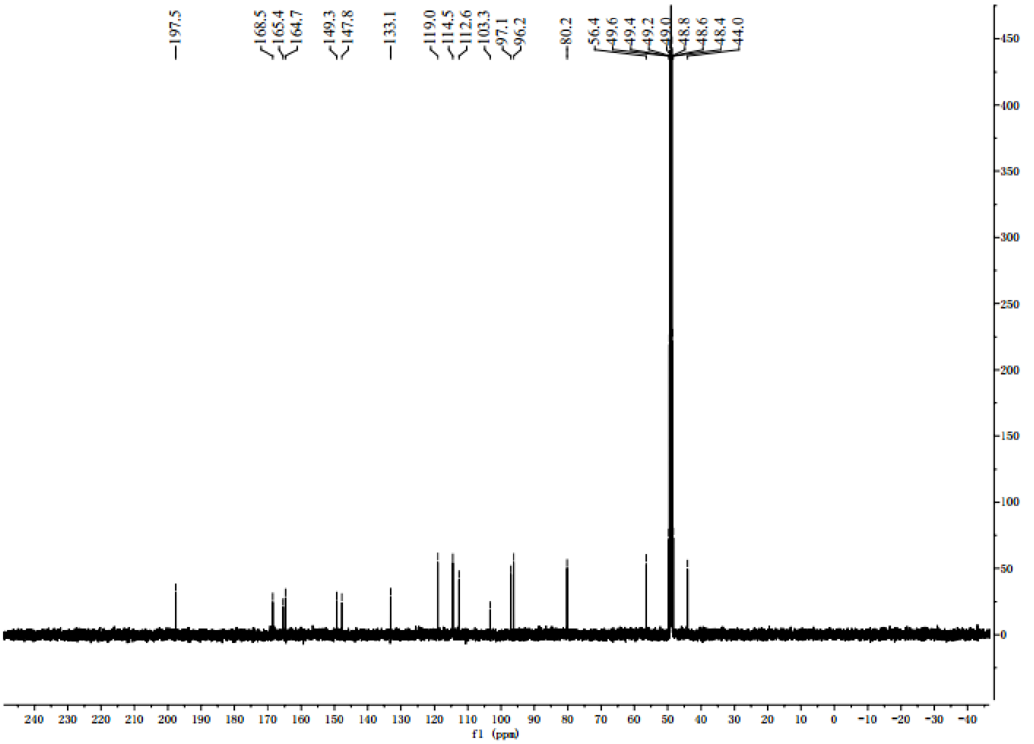

Figure S39. ^{13}C-NMR of Compound 17 (100MHz, CD_3OD)

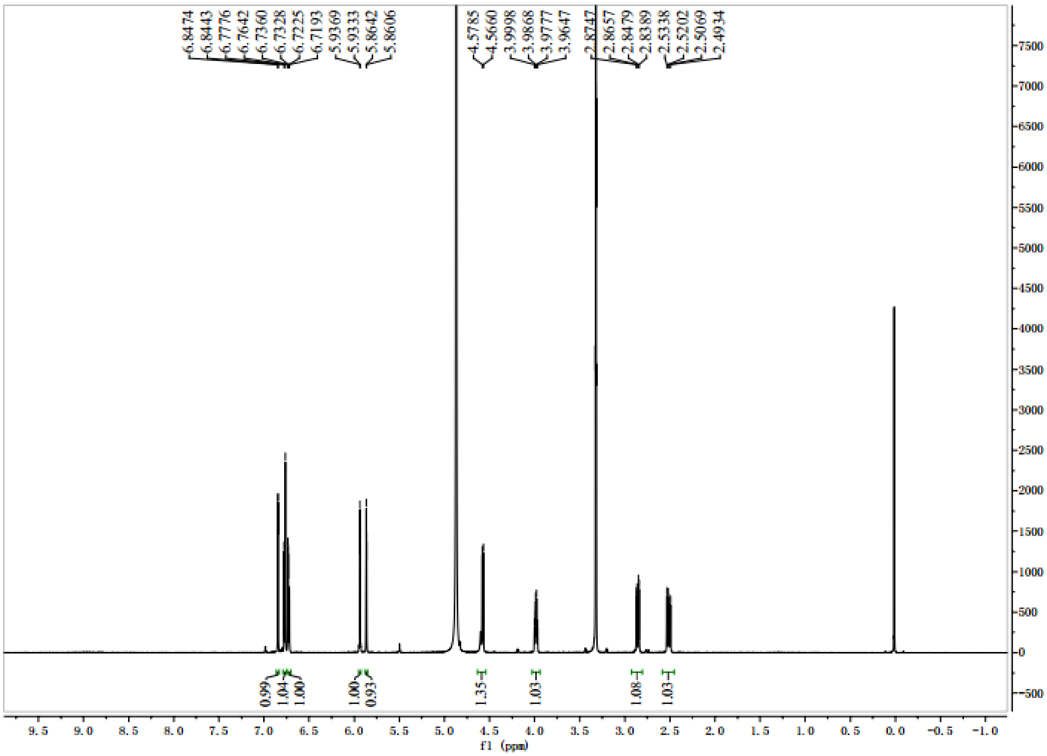

Figure S40. ^{1}H-NMR of Compound 18 (400MHz, CD_3OD)

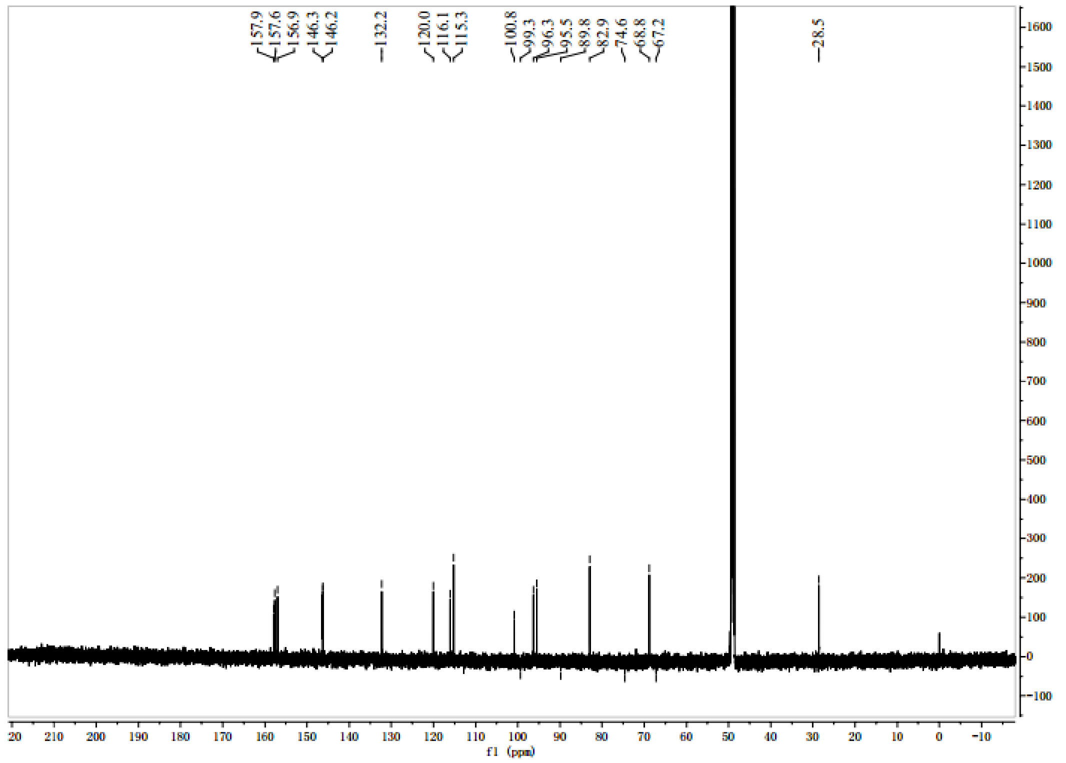

Figure S41. ^{13}C-NMR of Compound 18 (100MHz, CD_3OD)

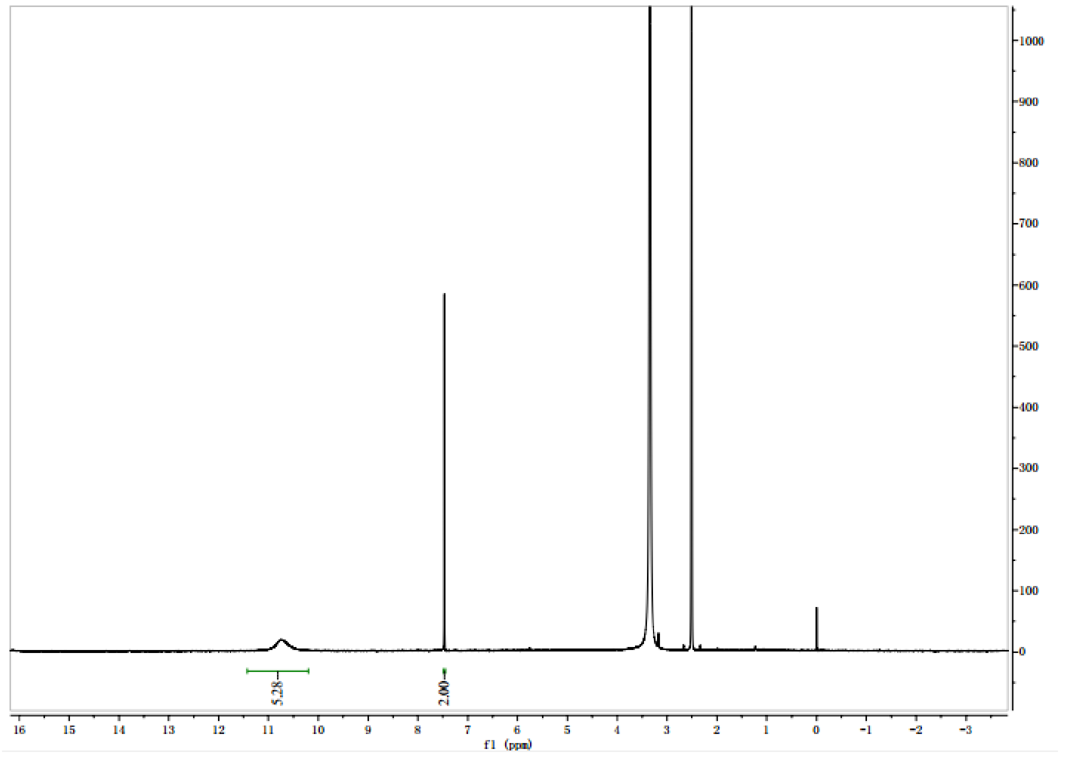

Figure S42. ^{1}H-NMR of Compound 19 (400MHz, DMSO-d_6)

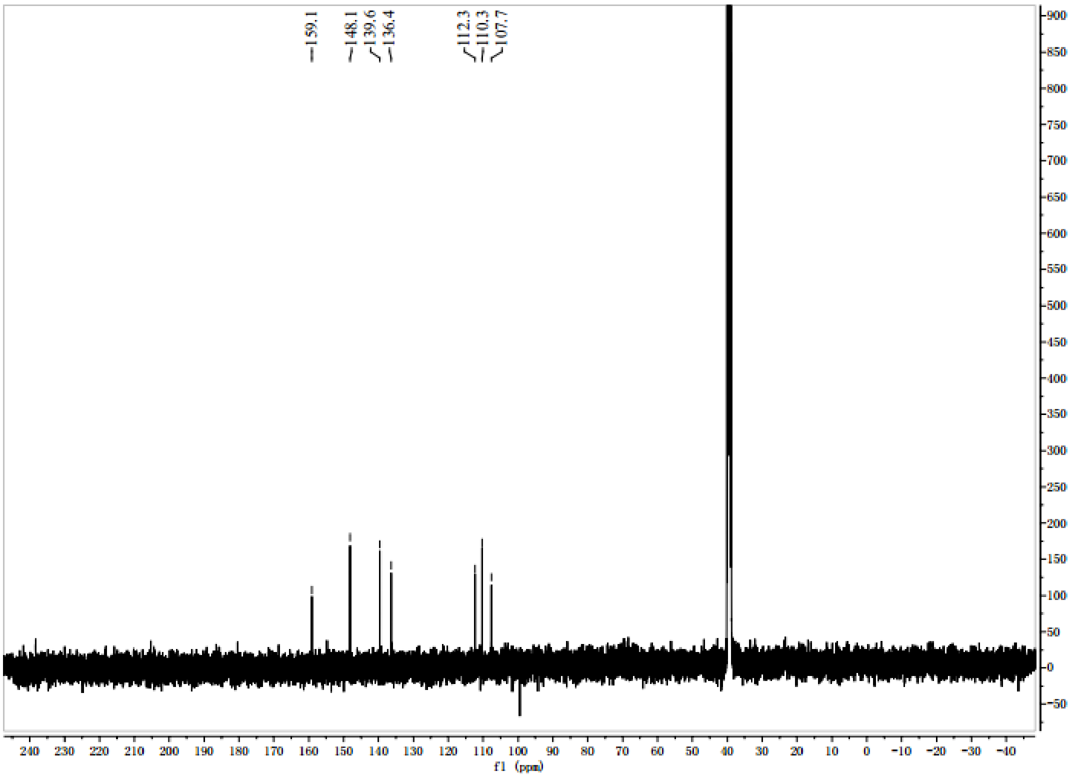

Figure S43. ^{13}C-NMR of Compound 19 (100MHz, DMSO-d_6)

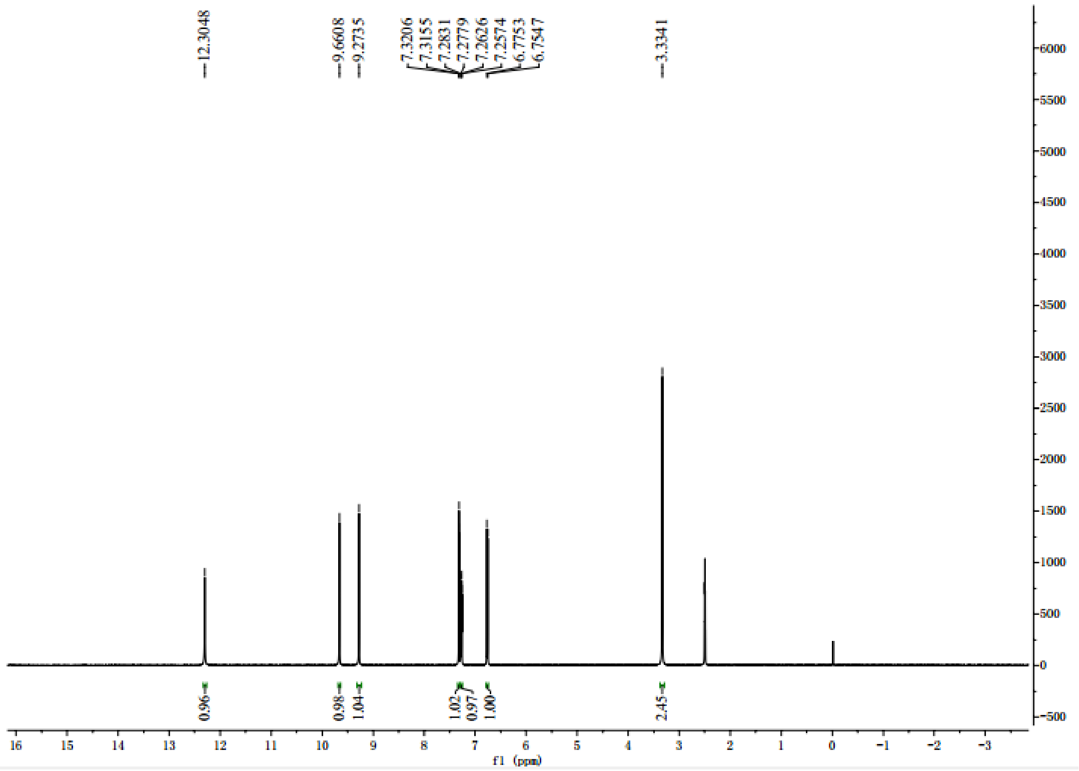

Figure S44. ^{1}H-NMR of Compound 20 (400MHz, DMSO-d_6)

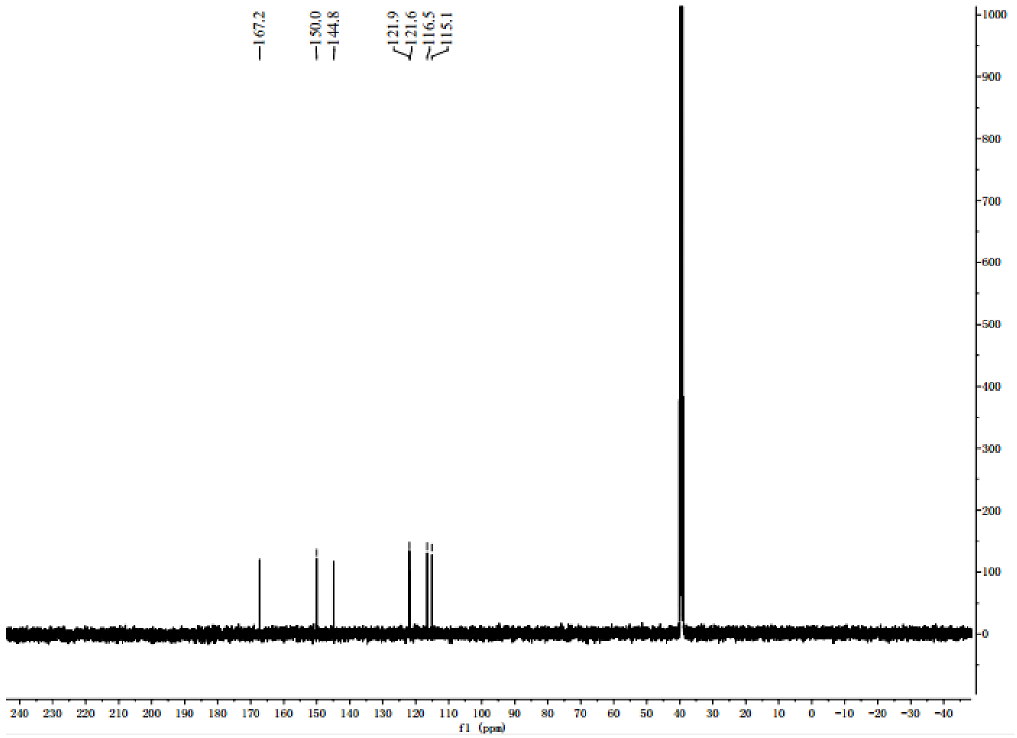

Figure S45. ^{13}C-NMR of Compound 20 (100MHz, DMSO-d_6)

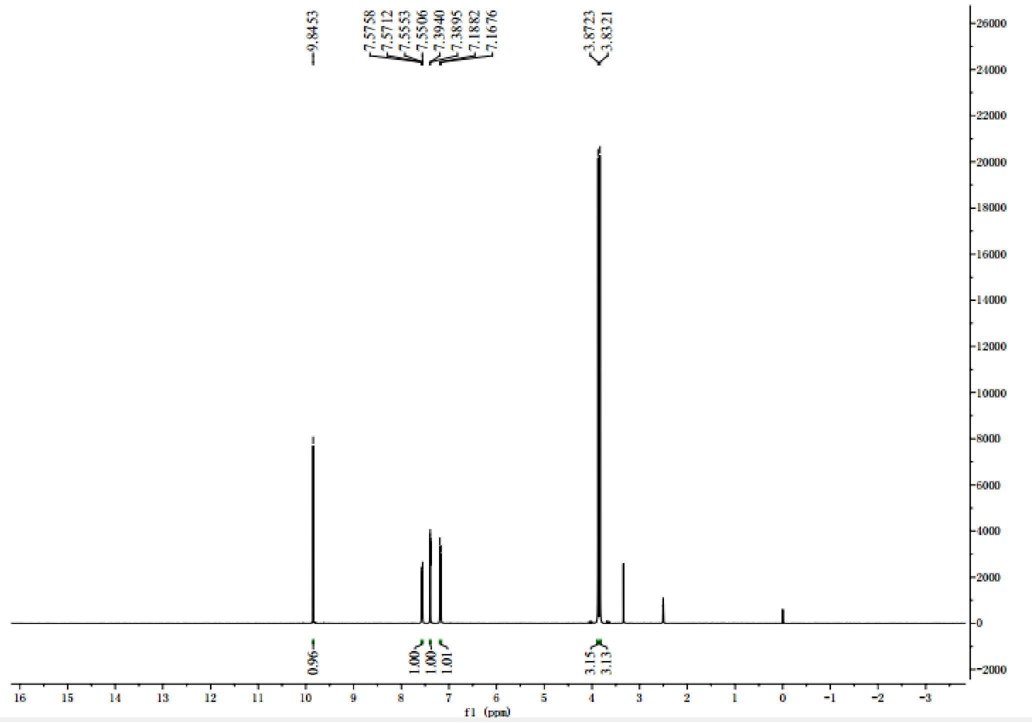

Figure S46. ^{1}H-NMR of Compound 21 (400MHz, DMSO-d_6)

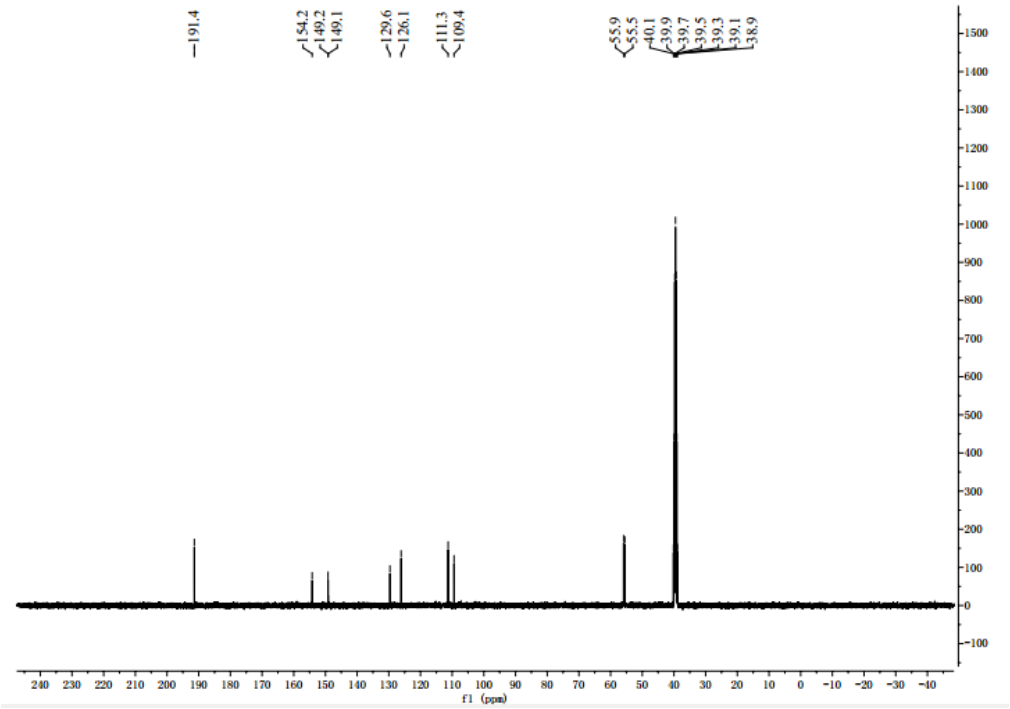

Figure S47. ^{13}C-NMR of Compound 21 (100MHz, DMSO-d_6)

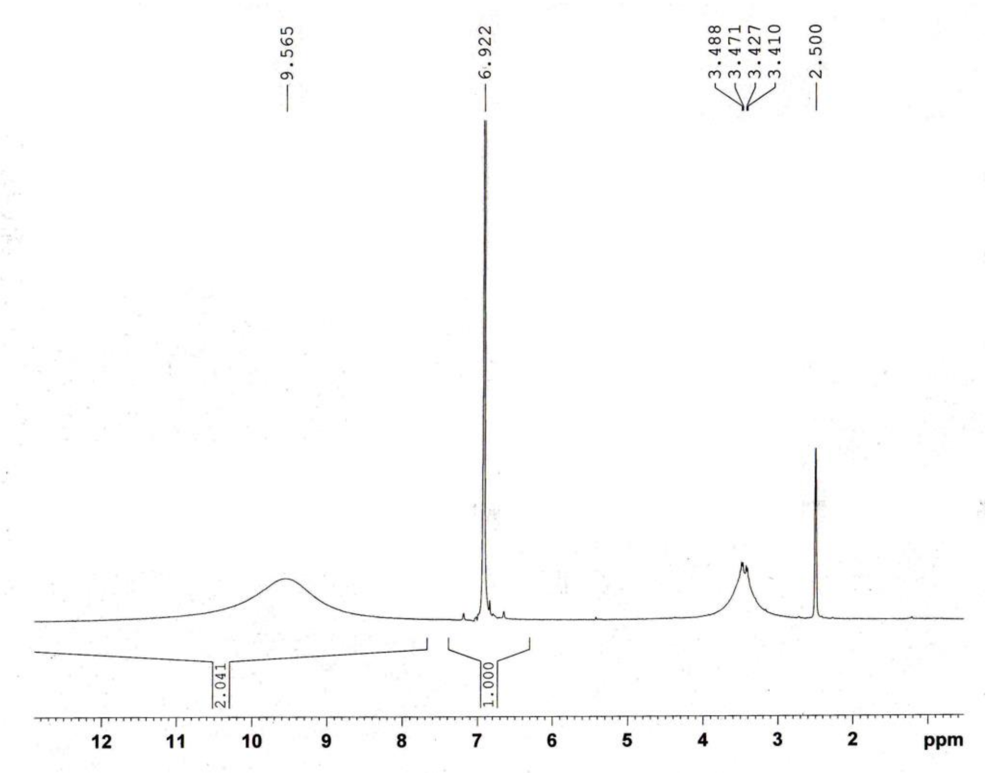

Figure S48. ^{1}H-NMR of Compound 22 (400MHz, DMSO-d_6)

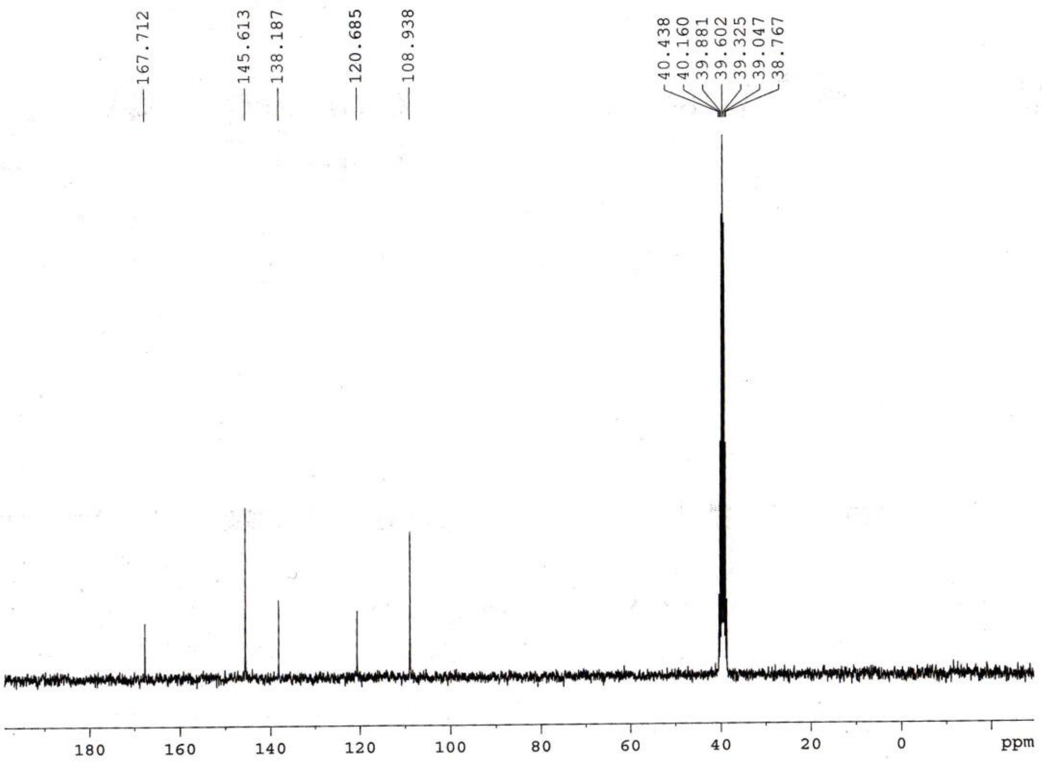

Figure S49. ^{13}C-NMR of Compound 22 (100MHz, DMSO-d_6)

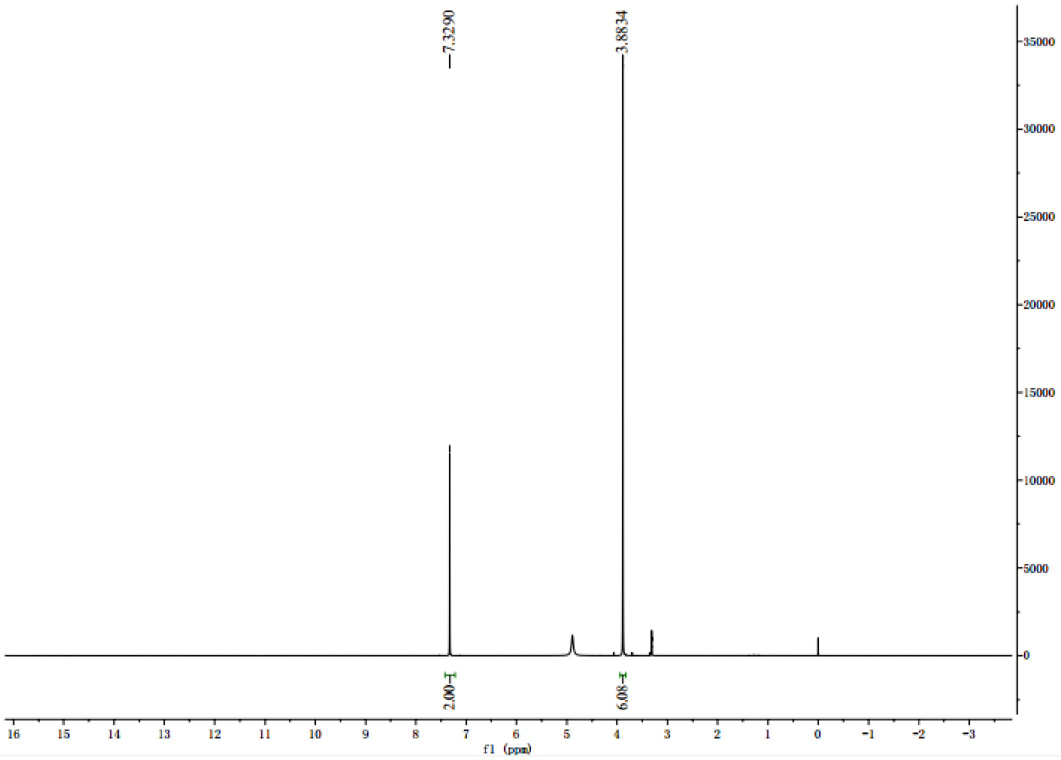

Figure S50. ^{1}H-NMR of Compound 23 (400MHz, CD_3OD)

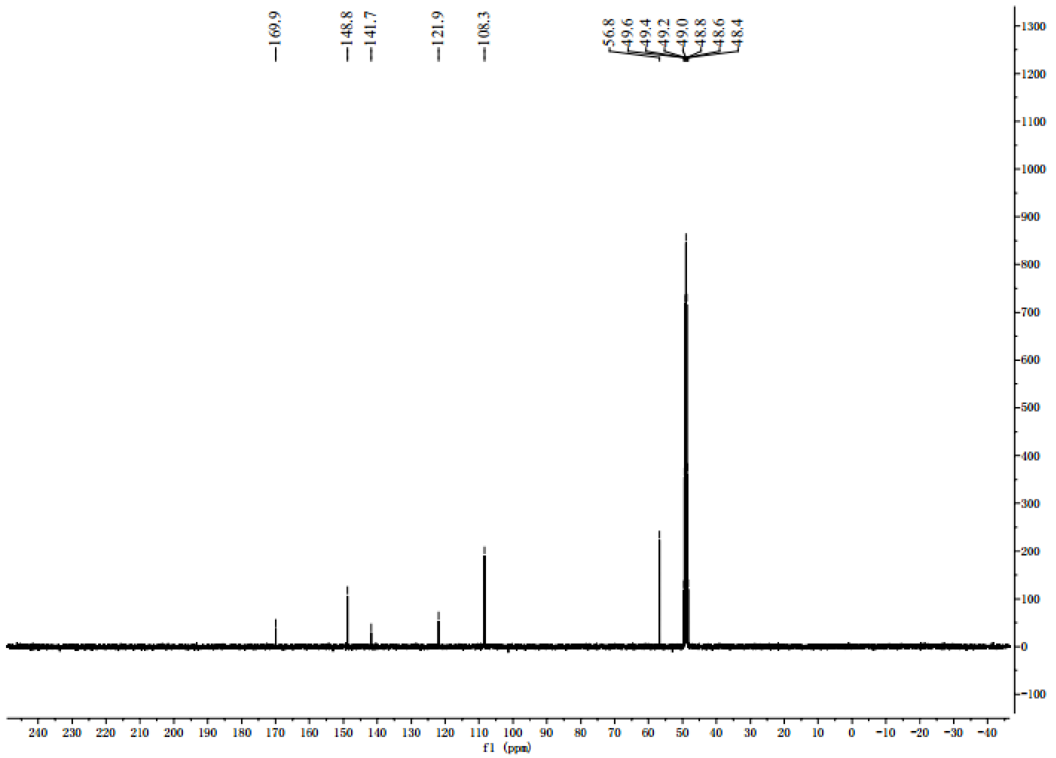

Figure S51. ^{13}C-NMR of Compound 23 (100MHz, CD_3OD)

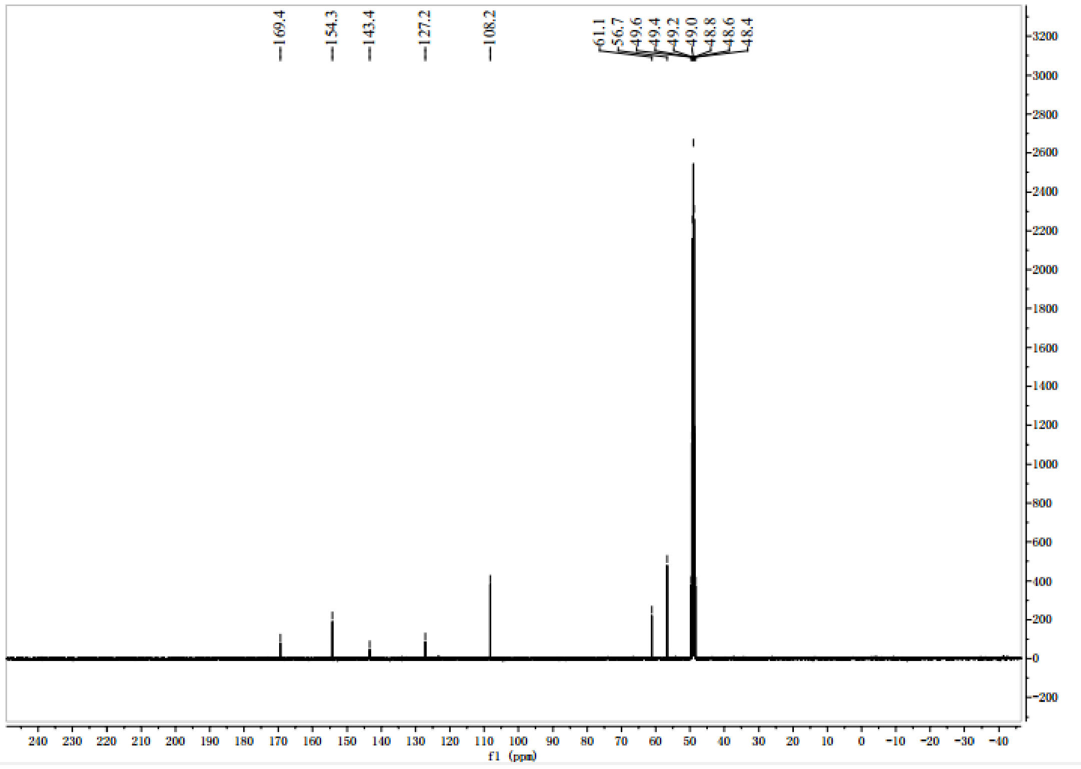

Figure S52. ^{13}C-NMR of Compound 24 (100MHz, CD_3OD)

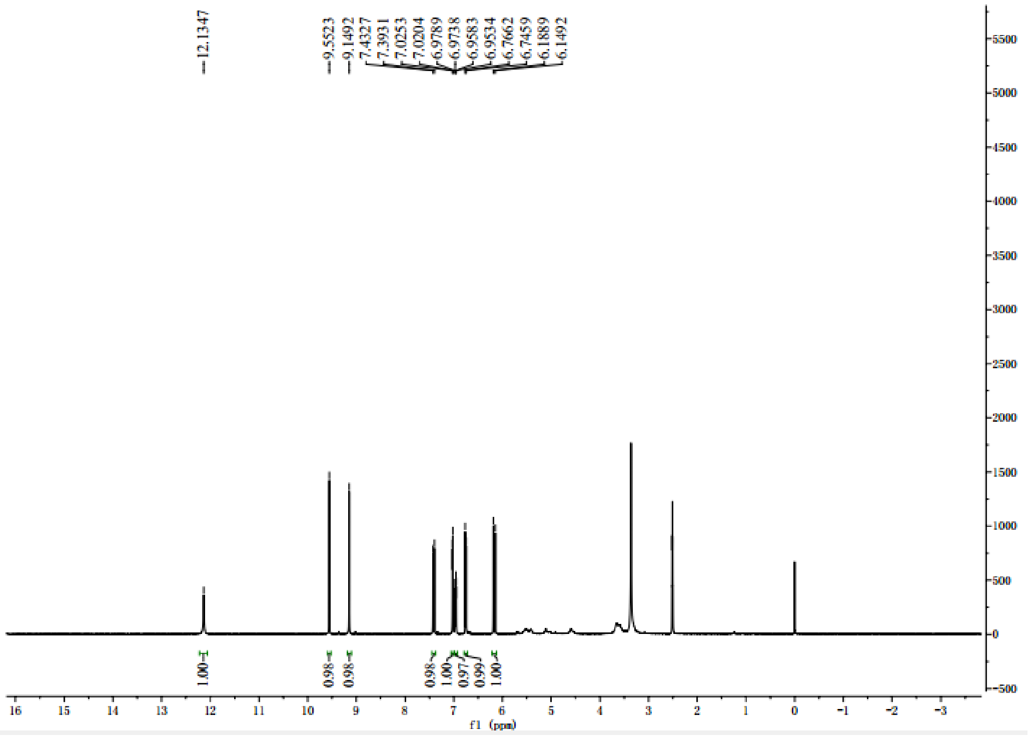

Figure S53. ^{1}H-NMR of Compound 25 (400MHz, DMSO-d_6)

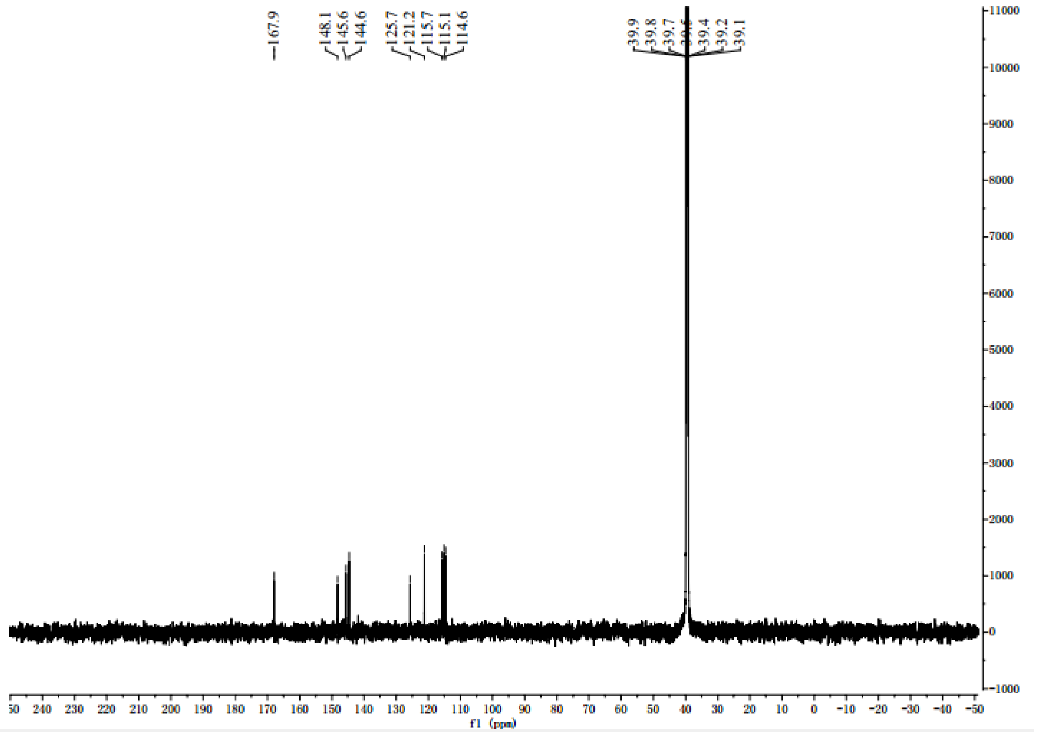

Figure S54. ^{13}C-NMR of Compound 25 (100MHz, DMSO-d_6)

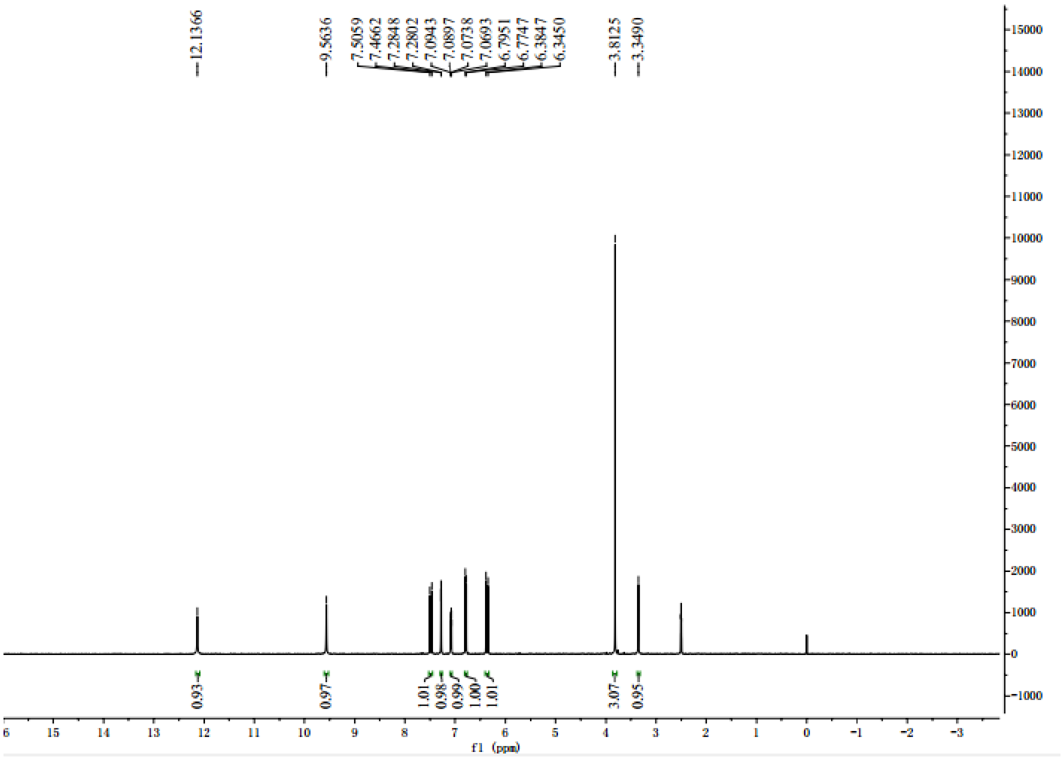

Figure S55. ^{1}H-NMR of Compound 26 (400MHz, DMSO-d_6)

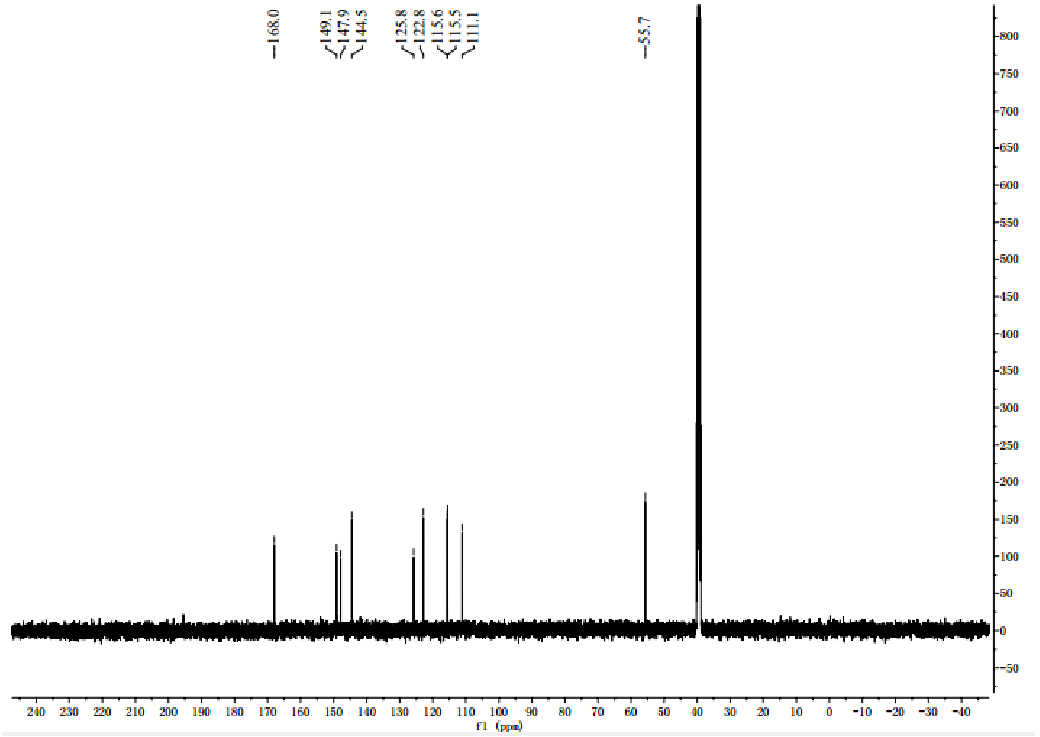

Figure S56. ^{13}C-NMR of Compound 26 (100MHz, DMSO-d_6)
